# Particles emitted from smouldering peat: size-resolved composition and emission factors†

**DOI:** 10.1039/d4ea00124a

**Published:** 2025-01-20

**Authors:** Amy L. Wilson, Wuquan Cui, Yuqi Hu, Marta Chiapasco, Guillermo Rein, Alexandra E. Porter, Geoff Fowler, Marc E. J. Stettler

**Affiliations:** a Department of Civil and Environmental Engineering, Imperial College London London UK m.stettler@imperial.ac.uk; b Department of Mechanical Engineering, Imperial College London London UK; c Sichuan Fire Research Institution of Ministry of Emergency Management of China Chengdu China; d Department of Materials, Imperial College London London UK

## Abstract

Peat fires emit large quantities of particles and gases, which cause extensive haze events. Epidemiological studies have correlated wildfire smoke inhalation with increased morbidity and mortality. Despite this, uncertainties surrounding particle properties and their impact on human health and the climate remain. To expand on the limited understanding this laboratory study investigated the physicochemical characteristics of particles emitted from smouldering Irish peat. Properties investigated included number and mass emission factors (EFs), size distribution, morphology, and chemical composition. Fine particles with a diameter less than 2.5 μm (PM_2.5_), accounted for 91 ± 2% of the total particle mass and the associated mass EF was 12.52 ± 1.40 g kg^−1^. Transmission electron microscopy imaging revealed irregular shaped metal particles, spherical sulfate particles, and carbonaceous particles with clusters of internal particles. Extracted particle-bound metals accounted for 3.1 ± 0.5% of the total particle mass, with 86% of the quantified metals residing in the fraction with a diameter less than 1 μm. Redox active and carcinogenic metals were detected in the particles, which have been correlated with adverse health effects if inhaled. This study improves the understanding of size-resolved particle characteristics relevant to near-source human exposure and will provide a basis for comparison to other controlled and natural peatland fires.

## Abstract 2

Peat fires emit large quantities of particles and gases, which cause extensive haze events. This study reports on the physicochemical properties of particles emitted from smouldering peat fires.

## Graphical abstract

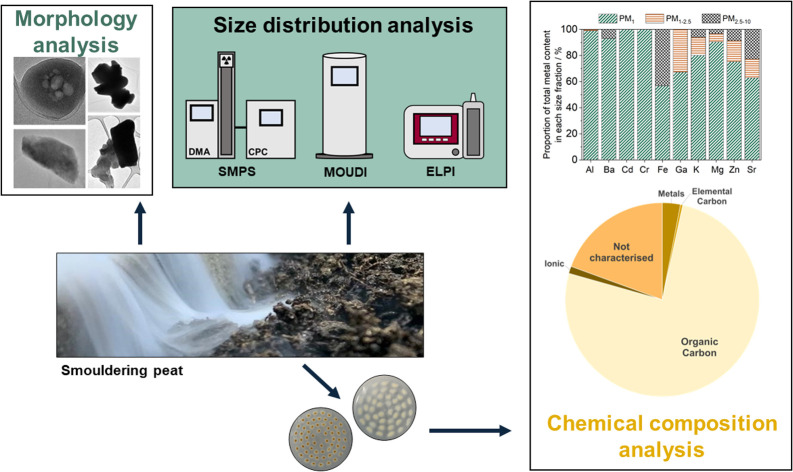

**Environmental significance**

Peat fires are some of the largest wildfires on Earth and their emissions lead to extensive regional haze events. Knowledge of the emitted particles' physicochemical properties in relation to climate forcing characteristics, and the causal mechanisms for adverse health effects, remains limited. In this study, emission factors (mass- and number-weighted) and the size-resolved chemical composition of particles emitted during laboratory peat smouldering fires were evaluated. The results find high levels of particle pollution, alongside metals being more abundant in the fine particle fraction that the coarse fraction. This may be of concern for regional air quality. This study provides peat fire emission characteristics for the use in future work on particle exposure health effects and atmospheric modelling studies.

## 1 Introduction

Annual increases in the Earth's surface temperatures and extreme weather events have been linked with the escalation of wildfires in America, central Asia, and southern Europe.^1–4^ One of the most vulnerable wildlands to fire, when drained for agricultural purposes or because of global warming, is peatland.^5,6^ Peat is a heterogeneous mixture of slowly decomposing plant material that accumulates in an anaerobic water-saturated environment.^7,8^ Peatlands cover around 3% of global land area whilst storing nearly a third of the territorial carbon.^9,10^ The fires can have both flaming and smouldering combustion dynamics, with the predominant form being smouldering. Smouldering fires are slow, flameless forms of burning that involve drying, pyrolysis and oxidation of the generated char phases.^11–13^ Peat fires can consume more than two orders of magnitude greater fuel than flaming fires per surface area burnt, and burn for extensively long burn periods, often lasting many days or months.^1,7,14^

Literature, to date, has focused on the gaseous emissions from peat wildfires.^15^ The wildfires are known to emit hazardous air pollutants, such as formaldehyde, benzene, and polyaromatic hydrocarbons (PAHs).^16–18^ Global carbon dioxide (CO_2_) emissions from peat wildfires is equivalent to more than 10% of the global anthropogenic CO_2_ emissions per year.^10,19,20^ Particles are emitted directly from peat wildfires and are also formed through gas-to-particle conversions in the smoke plume.^15^ However, research on the toxicity and physicochemical characteristics of the particles emitted from peatland wildfires is limited. There are only a few ambient (inclusive of field *in situ* and *ex situ* measurements, and aircraft campaigns)^21–31^ and laboratory studies that evaluate the particle emissions from the smouldering of peat.^18,32–37^ Therefore, the causal relationship between the particle properties and the health consequences of inhalation are largely unknown, even though studies have indicated fire haze events can result in increased regional hospital attendance, acute bronchitis, morbidity and mortality.^38–40^

Monitoring peatland fires in the field provides representative measurements but can be challenging due to remote locations, the size of the burns, and highly variable environmental conditions. Additionally, capturing the fire dynamics requires extensive monitoring periods. Proxies for the combustion dynamics, such as the modified combustion efficiency (MCE – the ratio of CO_2_ emitted to the sum of carbon monoxide (CO) and CO_2_) are not consistently reported and the peat mass loss rate (MLR) is not quantifiable in field studies.^15^ Field studies can also be impacted by the sampling environment; specifically, aspects such as particle aging within the plume.^41^ Consequently, the ability to compare emissions between field studies is limited.

In contrast, controlled laboratory experiments enable the isolation and investigation of specific peat combustion variables, such as moisture content, burn temperature and inorganic content, and their impact on fire dynamics and emissions.^18,42–47^ Laboratory experiments have also provided frameworks for the peat smouldering progression which include ignition, growth, steady, and burn-out stages.^18^ The combustion stages are determined through the monitoring of CO_2_ and CO emissions, temperature profiles, and MLR: the measurements show very little variation during the steady stage of the fire.^18^ Correlating the combustion dynamics with the concentration and composition of the emitted gaseous species and particles is important to understand and compare the toxicity of peatland wildfires across the world.

Furthermore, the particle size distribution (PSD), particularly in terms of particle mass and number emissions, is an important parameter for governing the particle deposition and clearance mechanisms in the body, as well as the inflammation and cardiac effects of the emitted particles.^48–50^ Particle number metrics alongside mass metrics are needed because ultrafine particles (UFP, particles less than 100 nm in diameter) contribute very little to the particle mass concentration (PMC), whilst they do contribute significantly to the total particle number concentration (PNC).^51^ Particles in the ultrafine range can deposit deeper in the lungs than coarse particles and can cross the air–blood barrier,^52^ which may lead to varying biological responses in comparison to exposure to particles with diameters less than 2.5 and 10 μm (PM_2.5_ and PM_10_).^48,49,53–56^ Additionally, *ex vivo* and epidemiological studies have correlated the UFP fraction emitted from smouldering fires with pro-inflammatory responses, increased reactive oxygen species production, and decreased cardiac function.^48,57^

The mass- and number-weighted PSD depend on the biomass type and the combustion dynamics,^37,58–60^ however the existing data is still very limited. Literature for peat fires has predominantly focused on the mass concentration of PM_2.5_, rather than considering the PSD.^22,32,35,61,62^ To date, number-weighted PSD and number emission factors (EF_N_) have not been extensively measured for peat fires compared to mass-weighted EFs (the particle number or mass emitted per unit mass of dry fuel that is burnt).^15,22,24,25,32^ Number concentrations were previously presented for ambient particle emissions from a peatland fire, but the combustion dynamics were unknown.^62^

Previous wood burning literature has found that smouldering combustion dynamics, or slower burning, led to a smaller number concentration of particles with a diameter less than 1 μm (PM_1_) than for higher temperature or faster burning fires.^59,60,63^ The opposite correlation was observed for mass concentration, a higher PMC was found for smouldering fires.^59,60,63^ Consequently, to understand the health risks associated with the full range of particles emitted from smouldering-only fires, number and mass metrics should be presented together.^48,50,53^

Additionally, there are only a limited number of studies that evaluate number-weighted PSD measured using different instruments. The existing research highlights instrument consistencies for reference aerosols, but variability for more complex particles.^64,65^ Particle bounce, particle de-agglomeration, and impactor overloading, as well as the various instruments having different sizing principles, have all been stated as reasons for the disparities.^66–68^ Comparison studies for complex particles, including those from peat burning, are therefore required to improve the confidence in particle number weighted data and to inform World Health Organisation (WHO) air quality guidelines.^69^

Studies considering the size-resolved chemical composition of particles emitted from peat fires are limited, but this is vital for understanding particle deposition, removal pathways, and the toxicity of inhaled smouldering particles.^57,70^ Laboratory and *in situ* field studies report that organic carbon (OC) is the predominant component of particles emitted from peat fires, ranging from 58 to 89% of the total particle mass.^24,32,35,71^ Source apportionment studies also state higher OC concentrations during haze events than for background measurements.^30,62^ A series of OC compounds and PAHs have been detected in particle emitted from peatland fires, and exposure to these compounds at concentrations above the WHO guidelines may result in health risks.^24,29,32^ Other OC compounds, such as levoglucosan, *n*-alkanes, and organic acids have been analysed for the purposes of source apportioning ambient particles, or to identify a biomarker for peatland fires.^24,30,48,62,72^ Metals and ionic species, such as potassium, aluminium, iron, chromium, nitrate, and sulfate have also been detected and quantified for PM_2.5_.^22,29,62,72,73^ Bio-available metals, such as Fe, can catalyse the formation of reactive oxygen species, which can induce a variety of inflammatory responses such as cardiovascular diseases.^74–76^ Additionally, a previous health risk assessment identified that four or five individuals out of 1000 may be at risk of cancer when exposed to the carcinogenic metals (such as chromium and nickel) in PM_2.5_ from peat fires.^29^ Health risk assessments have also highlighted the need to consider total, water-soluble, and extractable particle-bound transition metals (in various leaching agents) to understand the health consequences.^77^ Thus, additional laboratory-controlled research on the physicochemical composition of the particles emitted from smouldering peat fires is needed to evaluate the possible health implications for inhalation of fresh particles, by taking into consideration the particle's size, composition, and resulting toxicity.

To investigate the toxicity of the particles emitted from peat fires, this study used controlled laboratory experiments to quantify the size-resolved physicochemical properties of particles emitted during smouldering of horticultural Irish peat. The objectives of the study were to: (i) determine the number- and mass-weighted size distributions and total concentration of the particles emitted, (ii) quantify the particle number and mass EFs, (iii) quantify the concentration of organic and elemental carbon, metals, and ionic species in the emitted particles, and (iv) evaluate particle morphology using transmission electron microscopy. Particle size distribution, morphology, and chemical composition were of particular interest because these factors can influence the site of deposition in the lung, and the toxicity of the particles.^78–80^ Further details that aren't included in the main text can be found in the ESI,† where referenced.

## 2 Materials and methods

### 2.1 Peat samples

A commercial peat (Shamrock Irish horticultural moss peat, Bord na Mona Horticulture Ltd) was used because of its uniform properties and consistency between batches which ensured experimental reproducibility.^81^ The moisture content (MC, mass of water divided by dry peat mass) varies greatly in peatlands and is the single most important factor for ignition.^12,82^ Therefore, the MC was maintained at 100%, using a previously described protocol,^18,81^ to represent natural conditions whilst also ensuring the successful ignition of the peat. The peat density was 300.0 ± 7.5 kg m^−3^, and the carbon/hydrogen/nitrogen fractions of the dry peat were 50.21 ± 1.36%, 5.14 ± 0.18%, 1.65 ± 0.82%, respectively.^18,83^

### 2.2 Smouldering peat fires

**Fig. 1:**
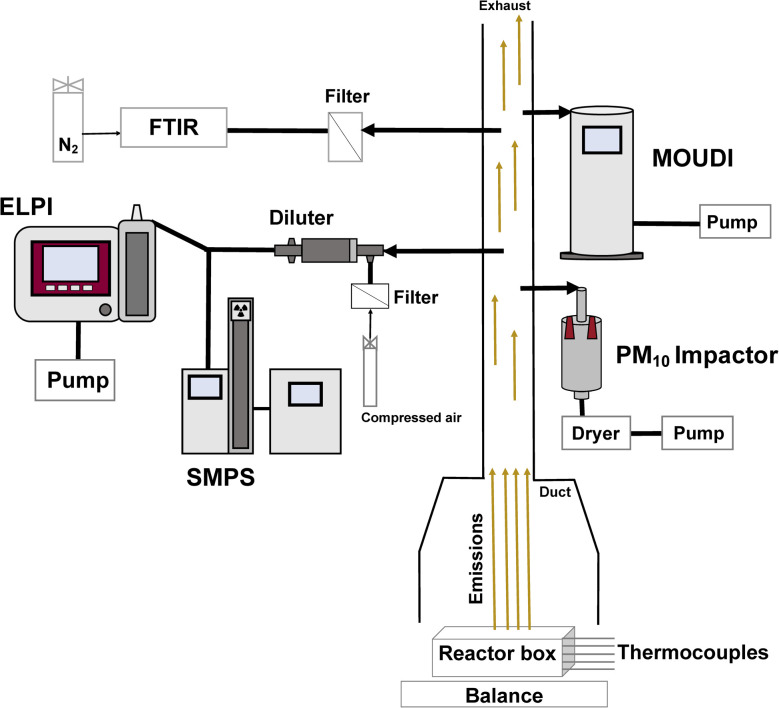
Schematic of the smouldering peat fire experiment and the instruments used to collect particles by cascade impaction and take real-time measurements of the particle number-weighted size distribution. Note, this schematic does not include diagnostics used for measuring the combustion dynamics.^18^

The peat samples were burned in an open reactor under controlled conditions, as shown in Fig. 1 and previously outlined in literature.^18,44^ Briefly, the sample was ignited using a heated coil along one side of the reactor at 5 cm depth and applying a 100 W power source to the coil until the mass measured by the balance decreased by 10%. The emissions were collected using an inverted fume extraction hood and were transported into a duct with a fan-controlled flow rate (0.034 m^3^ s^−1^ ± 2.5%). Four different diagnostic tools were taken during the experiments to monitor the smouldering dynamics: peat mass loss (resolution of 0.01 g), peat temperature profile (twelve K-type thermocouples), infrared imaging of the fire surface spread (FLIR Camera), and real-time concentration of 20 different gaseous species (Nicolet iG50 Fourier-transform infrared spectrometer, Thermo Scientific). The gaseous emissions have previously been reported.^18^

The experiment protocol was repeated 12 times to quantify various particle physicochemical properties (see Tables 1 and S-1 in the ESI,†) and the repeatability of the particle emissions. The combustion dynamics and particle emission results will be presented as the mean of the experiment measurements, with uncertainty quantified as the standard error of the experiment results. A comprehensive description of the particle measurements, collection procedures, and offline chemical analysis is provided below.

**Table 1:** Summary of particle collection methods and the physicochemical analysis undertaken to determine the particles' characteristics

Instrument or sampler	Particle size range/μm	Particle sample physical analysis	Chemical analysis technique	Chemical analysis
Scanning mobility particle sizer (SMPS)	0.016–0.550	PNC & PSD		
Electrical low-pressure impactor (ELPI)	0.006–10 (14 stages)	PNC & PSD		
Micro-orifice uniform distribution impactor (MOUDI)	0–9.9 (13 stages)	PMC & PSD		
Dekati PM_10_ impactor	0–10 (3 stages)	PMC & PSD	Ion chromatography (IC) and inductively-coupled plasma-optical emission spectroscopy (ICP-OES)	Ionic, low molecular weight acids, and elemental composition
Thermophoresis sampler	All particles (1 stage)	Morphology	Energy-dispersive X-ray spectroscopy (EDS) and Raman spectroscopy	Elemental composition
Filter collection using sampling pump	All particles		Organic carbon/elemental carbon instrument	Total, organic, and elemental carbon

### 2.3 Combustion dynamics

The combustion dynamics for the laboratory peat smouldering experiment comprise four stages of burning: ignition, growth stage, steady stage, and burn out.^18,84^ An example of the combustion dynamic profiles, including MLR, CO_2_ and CO, is presented in Fig. S-1 in the ESI.† During the ignition stage the mass loss rate increased to a maximum (0.03–0.04 g s^−1^), before declining in the growth stage (as the peat fire propagates away from the coil) to 0.02 g s^−1^. Between 20% and 80% sample mass loss (known as the steady stage, when the mass loss rate is ∼0.02 g s^−1^), the laboratory fire propagation is the most representative of smouldering fire spread in the field and, unlike the ignition phase, is least affected by the ignition coil.^9,44^ For these reasons, only the results from the steady stage are presented in this paper.

Table 2 includes data used to monitor combustion dynamics during the steady stage. The average MCE value was consistent with literature values for smouldering fires (MCE – 0.65 to 0.85), and below the value for flaming-only fires (0.99).^15,41,47^ The average peak temperature and spread rate were also consistent with other smouldering studies; smouldering combustion is low-temperature, peaking at around 500 to 700 °C, and slow spreading (1 to 3 cm h^−1^) relative to flaming combustion of peat, where temperatures reach 1500 to 1800 °C and spreading is two orders of magnitude faster.^7^

**Table 2:** Average combustion dynamic parameters for the smouldering Irish peat experiments

Peat	Spread rate/cm h^−1^	Mass loss rate/g s^−1^	Modified combustion efficiency	Peak temperature/°C
Irish (H)	1.17 ± 0.11	0.0211 ± 0.0022	0.860 ± 0.011	578.93 ± 36.30

### 2.4 Particle concentration and size distribution

Two real-time measurement instruments were used to determine the particle size distribution and number concentration. The Scanning Mobility Particle Sizer (SMPS, Model 3938, TSI Inc) and the Electrical Low-Pressure Impactor (ELPI+™, Dekati Ltd) were used in parallel, as shown in Fig. 1, to measure the particle size distributions between 6 nm and 10 μm. An ejector diluter (L7 Diluter, Dekati Ltd) was placed in line with the duct prior to the SMPS and ELPI, to dilute the air by 8 : 1.

The SMPS consisted of a long Differential Mobility Analyser (DMA, Model 3081A, TSI Ltd), a Condensation Particle Counter (CPC, Model 3756, TSI Inc), and a neutraliser (Model 3088, TSI Ltd). It was operated with a sheath flow of 3 L min^−1^ and the CPC flow rate at 0.3 L min^−1^. Particles with diameters between 16 nm and 560 nm were separated according to their electrical mobility diameter (*d*_m_), and their number concentrations were corrected for multiple charge effects and diffusion loss using the in-built SMPS software algorithms. *d*_m_ is defined as the diameter of a unit density spherical particle moving at the same velocity in an electric field as the particle in question.^85^

The ELPI was operated at a flow rate of 9.66 ± 0.03 L min^−1^. The size distribution and number concentrations were collected every second for particles sized between 6 nm and 10 μm. The particles, which were charged by the corona charger, entered the low-pressure cascade impactor, were separated according to their inertia and collected on the 14 sintered plate stages. Particles were classified by their aerodynamic diameter (*d*_a_); the diameter of a unit-density sphere having the same gravitational settling velocity as the particle in question.^85^ Additional information on the calculation of the number concentration can be found in the ESI.†

A three-stage PM_10_ Impactor (Dekati Ltd) was used to collect particles for chemical analysis (ionic and metal content) using polycarbonate (25 mm, Nuclepore Track Etch Filter, Whatman) and borosilicate glass microfibers filters (47 mm, Emfab, Pallflex) using a 30 ± 1.5 L min^−1^ flow rate. The three-stages and back-up filter had cut-off points of 10 μm, 2.5 μm, and 1 μm, respectively. Filters were weighed (Sartorius BP211D Basic Plus Analytical Balance, 0.00001 g resolution) to determine the PMC.

A Micro-orifice Uniform Deposition Impactor (MOUDI, MSP, Model 125R) was also used to collect particles for gravimetric analysis using PTFE filters (Fluoropore, 0.45 μm pore size, 47 mm diameter) using a flow rate of 9.5 ± 0.3 L min^−1^. The thirteen-stages had cut-off points of 9.9, 6.2, 3.1, 1.8, 1.0, 0.56, 0.31, 0.18, 0.1, 0.055, 0.032, 0.018, 0.010 μm. The additional MOUDI stages, in comparison to the PM_10_ Impactor, provided additional insights into the particle mass-weighted size distribution, as well as the contribution from the UFP fraction.

The uncertainty in the particles mass measurements for the MOUDI and Dekati PM_10_ Impactor samples were estimated by propagating the standard deviation of the triplicate measurements of pre- and post-sampling filter mass and the standard deviation in the instruments flow rate, as presented in Tables S-2 and S-3.† The analytical uncertainty was less than the standard error, so we report the latter for all gravimetric and chemical analyses.

### 2.5 Morphology, particle size, and elemental composition

Particles were collected on 300-mesh copper transmission electron microscope (TEM) grids coated with a lacey carbon supporting film using a thermophoretic sampler set at 5 V (DC), with a flow rate of 0.3 L min^−1^.^86^

Particle size, shape, and the composition of 60 individual particles that had a Feret diameter of less than 10 μm were analysed using a Jeol 2100Plus Scanning TEM (STEM, Jeol Ltd, UK) coupled with an energy dispersive spectroscopy (EDS) instrument (X-max 80T Aztec detector, Oxford Instruments). The TEM was operated at 200 kV, whilst the elemental maps were acquired for 30 minutes with a 15° solid angle using EDS. Particles with a diameter greater than 10 μm were not analysed.

### 2.6 Particle chemical composition analysis

Extractable metals: a closed vessel microwave digestion system (Multiwave 3000, MF100 drum, Anton Parr) was used for the digestion and extraction of elements from the particle samples collected using the PM_10_ Impactor (cut-off points of 10, 2.5, and 1 μm). Prior to their use, the microwave vessels were soaked overnight in 10% nitric acid (HNO_3_) to remove any contamination. The microwave protocol was adapted from previously validated methods that are stated in literature.^87^ Briefly, half of each filter underwent HNO_3_ (68%) microwave digestion. The microwave was set to 3 min ramp to 95 °C, 5 min hold, 3 min ramp 120 °C, 5 min hold, 3 min ramp to 130 °C, and 2 min hold to extract the elements. A diluted extracted solution was filtered through a 0.45 μm cellulose membrane filter prior to being analysed by ICP-OES (Avio 500, PerkinElmer) for the determination of the concentrations of 18 elements: Al, B, Bi, Ba, Cd, Co, Cr, Cu, Fe, Mg, In, Ga, Mn, Ni, K, Sr, Pb, and Zn. The concentration of the metals is presented as an average of the nine collections with their associated standard error. The limit of detection and quantification for each element is shown in Table S-4.† Certain elements, such as B, Bi, Pb, Ni, In, Cu, Co, and Mn were not detected, or their concentration were below the quantification limit.

Particle ionic content was determined by Ion Chromatography (IC) (Dionex ICS-2100, Thermo Scientific). The ionic species were extracted from half of each filter (PM_10_ Impactor filters) using 10 mL of 18.2 MΩ ultrapure water and ultrasonic agitation for 1 h.^62^ The extract was subsequently filtered through a 0.45 μm cellulose membrane filter and analysed for K^+^, Ca^2+^, Mg^2+^, Na^+^, Li^+^, Br^−^, F^−^, Cl^−^, SO_4_^2−^, PO_4_^3−^, NO_3_^−^, NH_4_^+^, and NO_2_^−^, as well as three low molecular weight acids (oxolate, formate, and acetate). The concentrations of these ions are presented as a mean of the six collections with their associated standard error. The limit of detection and quantification for each element is shown in Table S-5.† Certain ions, including Br^−^, PO_4_^3−^, and Li^+^, were not detected.

Three blank filter samples, that had been placed in the PM_10_ Impactor prior to any experiments, were also analysed for ionic and metal content.

Organic and elemental carbon: particles were collected on quartz fibre filters (47 mm, Tissuquartz, VWR) for 20 minutes using an air sampling pump (Sidekick, Model 224-52MTX, SKC) at a flow rate of 2 L min^−1^. The National Physical Laboratory (NPL, UK) then analysed the bulk samples for OC and EC content by taking a 1.5 cm^2^ punch of the filter and subjecting the sample to thermal optical analysis using an OC/EC analyser (Organic Carbon/Elemental Carbon Instrument, Model 5 L, Sunset Laboratory Inc). This followed NPL's ISO 17025 accredited in-house procedure QPAS/B/561 using the EUSAAR2 thermal protocol. The uncertainty in the TC measurements is reported as the standard error multiplied 2 (∼95% confidence interval), in accordance with NPL's UKAS accreditation.

Particle phase was analysed by Raman Spectroscopy (inVia Raman Microscope, Renishaw). The instrument was equipped with confocal optics and a nitrogen-cooled charge coupled device (CCD) camera detector. The excitation laser beam (confocal mode at 532 nm) was focused on a sample area of ∼100 nm, with a final laser power of 0.5 mW (1% of laser power). The acquisition time was 10 s per accumulation, with a total of 5 accumulations per particle sample area. 18 particles were analysed to gain an insight into the sample structural heterogeneity.

### 2.7 Emission factor calculation

The carbon mass balance approach was used to determine the fuel-based CO_2_ EFs, in units of mass of analyte per kilogram of fuel burned, as shown in eqn (1).^88^1
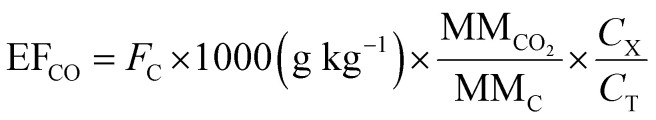
where *F*_C_ is the mass fraction of carbon in the dry biomass, MM_CO_2__ is the molar mass of CO_2_ (g per mole), MM_C_ is the molar mass of carbon (g per mole), *C*_X_ is the number of moles of emitted species (X) and *C*_T_ is the total number of moles of carbon emitted (including carbon in gaseous and particulate form).

The peat carbon content (*F*_C_ = 50.21 ± 1.36%) as well as the concentration of carbon detected in the particle and gaseous phases were included in the EF calculations.

The carbon content in the remaining residue after the smouldering experiments was measured using a CHNS elemental analyser (Flashsmart CHNS/O, CE Instruments Ltd). The fraction of carbon remaining in the residue, was found to be approximately 1.5% of the total unburnt peat carbon content. This value was included in the carbon mass balance calculation of the particle mass, particle number, and particle-bound species EFs, as shown in eqn (2).^36,71^2
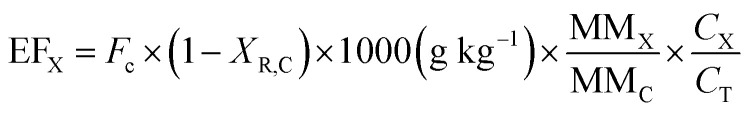
where *X*_R,C_ is the fraction of fuel carbon that remains in the residue after the burning of peat is complete (no additional mass is lost). CO_2_ was then used as a reference species to calculate the particle mass and number EFs, as well as the composition related EFs using the following equation.^24,89^3
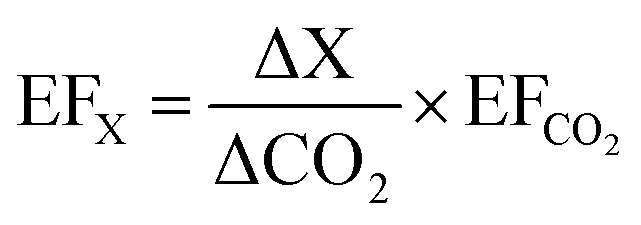
where ΔX is the concentration (mass concentration, mg m^−3^ and number concentration, cm^−3^) of the species X and ΔCO_2_ is the concentration of CO_2_ (mg m^−3^).

## 3 Results

The physicochemical characteristics including size distribution, concentration, morphology, and the chemical composition of particles emitted from the Irish peat smouldering fires were evaluated. The PM_2.5_ fraction accounted for 94 ± 2% of the total particle mass sampled, whilst particles with a diameter less than 560 nm accounted for more than 95% of the total particle number concentration. Particles were also collected for offline analysis of OC, EC, metal, and ionic content. The chemical mass balance was in reasonable agreement with the gravimetric analysis, with a mass deficiency of approximately 19% being attributed to elements not analysed for (such as Si), measurement errors, and water content. The particles emitted from the smouldering peat burns had a high carbon content. The OC mass fraction ranged from 74 to 78% of the total particle mass; however, the EC fraction accounted for less than 0.5% of the total particle mass. These values agree with laboratory and in-plume peat fire studies, including those from temperate, tropical and boreal peat fires, as shown in Table S-6 in the ESI.† The average total quantifiable metal mass accounted for 3.1 ± 0.5% of the total particle mass (PM_10_), in agreement with previous laboratory and ambient peat fire studies (range 0.07 to 13% of particle mass).^27,29,62,71,90,91^ The total quantifiable ionic mass contributed 1.2 ± 0.4% to the total particle mass (excluding ions which were characterised under the metal analysis), whilst the low molecular acids contributed approximately 0.6% of the total particle mass. This average ionic mass value was at the lower end of the range found in literature for particles emitted from peat fires (1–40%).^24,62,92^

The following sections will evaluate the size distribution and size-resolved chemical composition of the particles emitted from the smouldering peat fires.

### 3.1 Particle mass distribution

**Fig. 2:**
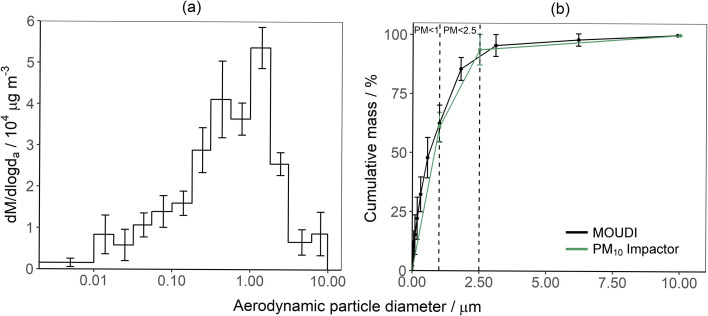
(a) The average mass-weighted size distribution of particles collected using the MOUDI during the steady stage of burning (20 to 80% of the dry peat mass lost). (b) The average cumulative mass of the particles collected using the PM_10_ Impactor and the MOUDI. Error bars represent ± standard error.

The average MOUDI-measured mass-weighted particle size distribution of emitted particles from the smouldering fires is given in Fig. 2, alongside the percentage cumulative mass calculated for both the MOUDI and PM_10_ Impactor measurements. We observe two peaks in the mass-weighted particle size distribution, between 0.32 to 0.55 μm and 1 to 1.8 μm, which is in agreement with previous literature.^92^

The PM_10_, PM_2.5_, and PM_1_ mass concentrations are shown in Table 3. The PM_2.5_ mass concentrations, measured by the MOUDI and PM_10_ impactor, were equivalent to 91 ± 2% and 94 ± 2% of the total particle load, respectively. The large contribution of the PM_2.5_ fraction to the total PM_10_ mass is consistent with existing literature.^15,18,25,48,61^ The PM_1_ fraction accounted for 69 ± 4% and 65 ± 1% of the PM_2.5_ fraction, for the samples taken using the MOUDI Impactor and the PM_10_ Impactor, respectively.

**Table 3:** Average mass concentration and emissions factors collected using the PM_10_ Impactor and MOUDI. Errors represent ± standard error where *n* = 13 for PM_10_ Impactor measurements and *n* = 6 for MOUDI measurements

Particle fraction	Instrument	Mass concentration/μg m^−3^	Mass emission factor/g kg^−1^
PM_1_	PM_10_ impactor	4120 ± 567	8.16 ± 0.98
	MOUDI	4191 ± 761	8.86 ± 1.32
PM_2.5_	PM_10_ impactor	6304 ± 853	12.47 ± 1.44
	MOUDI	5884 ± 810	12.52 ± 1.40
PM_10_	PM_10_ impactor	6721 ± 916	13.34 ± 1.54
	MOUDI	6541 ± 1000	13.84 ± 1.62

The average PM_2.5_ EF_M_ were within the range of existing temperate peat fire measurements in the laboratory (6.6 to 49 g kg^−1^, *n* = 5).^18,25,32,33,36^ To date, only one field study in North Carolina,^25^ to the authors knowledge, has quantified PM_2.5_ EF_M_. The PM_2.5_ EF_M_ associated with the North Carolina study stated an EF_M_ approximately four times larger than this study's measurements for the combustion of Irish peat.

The combustion dynamics resulting from the variations and interactions between the peat density, MC, inorganic content, peat porosity, and carbon content, as well as the ignition protocols and diffusion of heat and oxygen through fuel, may explain the large variation of EF_M_ presented in the literature.^9,43,47,60,93^ Additionally, environmental factors such as the time since the last peat fire or disturbance to the peatland may impact the emissions from the fires.^32^

The differences between the EF_M_ measured by the MOUDI and PM_10_ Impactor for PM_1_, PM_2.5_, and PM_10_ fractions are within the measurement uncertainty. The small discrepancy for particles with a *d*_a_ < 2.5 μm may be a result of reduced collection efficiency potentially caused by particle bounce and internal losses within the PM_10_ impactor.^85^ Furthermore, the rotation of the MOUDI stages, as well as the micro-orifice nozzles, are expected to reduce overloading, minimise particle bounce and re-entrainment of particles, as well as minimising losses of semi-volatile and volatile particles.^94^ These additional features enabled longer collection times with the MOUDI compared to the PM_10_ Impactor, which enabled the MOUDI to capture more of the variability in the particle emissions over the course of the steady stage of the peat fires.

Nevertheless, due to the inhomogeneity in composition of *in situ* peat (often undisturbed) and the environmental factors impacting fresh particle emissions, these mass metrics cannot be scaled to natural peat fires without a consideration of the combustion dynamics, peat density, composition, and MC.

### 3.2 Particle number distribution

To date, inter-comparison studies that evaluate the measurement of mass- and number-weighted particle size distribution are limited. Specifically for peat fires, the studies that have measured the number-weighted particle size distribution have not evaluated the same particle size range, leading to large uncertainty when comparing results. This study addressed this by using two near real-time devices, which classify the particles based on different equivalent diameters, and comparing the number size distributions and EF_N_.

**Fig. 3:**
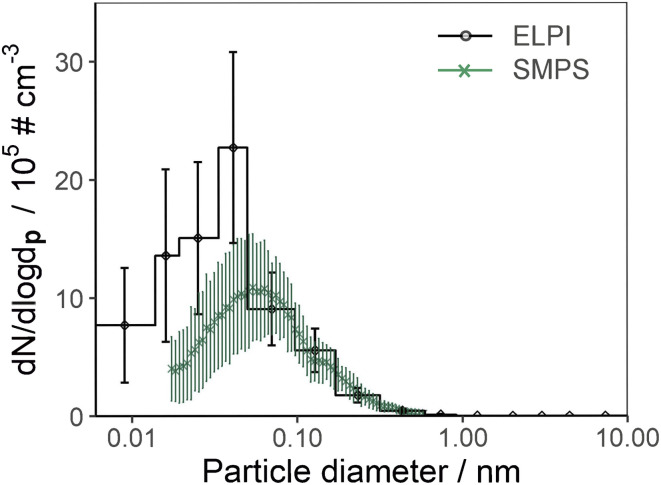
An example of the number-weighted particle size distribution for the ELPI and the SMPS measurements. Error bars represent ± standard error.

The normalised number-weighted particle size distributions from the ELPI and SMPS measurements are presented in Fig. 3. The SMPS measurements showed a bimodal distribution with peaks at 60 nm and 150 nm, similar to a previous ambient study,^92^ however a bimodal distribution is not consistently observed during biomass haze events.^28^

The average GMD for both instruments was in the UFP range (<100 nm), as shown in Table 4. The SMPS derived GMD was in agreement with the limited number of studies that considered the particle number-weighted size distribution (using mobility diameter as the equivalent diameter) for peat fires, as displayed in Table 4. The disparity between the aerodynamic and mobility diameter size distributions (*d*_a_ and *d*_m_) was confirmed by instrument checks that showed that the electrical currents measured by all ELPI stages were above the noise limits, all instrument parameters were correct, and appropriate zeroing methods had been followed. The particles emitted from the peat fires are complex in morphology (see Section 3.6), and the particle density is unknown and is likely to vary for particles of different composition. These factors all impact the charging mechanisms within the instruments and the equivalent diameter reported by the ELPI and SMPS.

**Table 4:** Comparison of the particle number emission data from this study and existing literature. For this study, error bars represent ± standard error where *n* = 5 for the SMPS and *n* = 7 for the ELPI measurements

Instrument (particle size range)	Location (peat)	Number concentration/10^5^ cm^−3^	Number emission factor/10^15^ kg^−1^	GMD/nm	Reference
SMPS (16–560 nm)	Laboratory (Irish)	6.57 (±1.43)	1.49 (±0.16)	74 (±4)	This study
ELPI (0.06–10 μm)		21.90 (±4.76)	4.61 (±0.71)	29 (±3)	This study
CPC (0.01–2.5 μm)	Ambient (Indonesian)	0.21–1.7			See *et al.* (ref. 62)
SMPS and APS (0.008 to 19 μm)	Ambient (Indonesian)	5.31 (±8.33)		Bimodal (50 nm and 400 nm)	See *et al.* (ref. 92)
Fast mobility particle sizer – FPMS (5.6–560 nm)	Ambient (Indonesian)	0.40 (±0.03)		60	Betha *et al.* (ref. 28)
SMPS (15–680 nm)	Laboratory (Florida)	5.38 (±4.17)	2.88 (±2.82)	90	Bhattarai *et al.* (ref. 37)
SMPS (15–680 nm)	Laboratory (Siberian)		6.86 (±3.93)		Bhattarai *et al.* (ref. 37)
	Laboratory (Malaysian)		3.14		

For example, the particle density may vary as a function of size and therefore, using a single particle density value in the ELPI calculations may not be suitable.^95,96^ As the *d*_a_ GMD was smaller than the *d*_m_ GMD, the emitted particles may have a density less than 1 g cm^−3^, less dense particles impact on lower stages in the ELPI instrument than an equivalent particle with unit density. Additionally, the particles are not all spherical, which has previously been found to lead to an ELPI size distribution that is broader than the SMPS distribution.^66,97^ A previous study also stated a potential mobility diameter peak less than 0.01 μm may exist for particles emitted from peat fires, but because of the size range of the SMPS the peak could not be resolved.^92^ Furthermore, the rapidly varying particle emission rates may have exacerbated the small particle correction of the ELPI.^95^ Consequently, having both the *d*_a_ and *d*_m_ measurements provides greater insight into the physical behaviour of the particles.

The smouldering average PNC and EF_N_ values for measurements with both the ELPI and SMPS are shown in Table 4 alongside existing literature values. This study's average PNCs, determined from the respective instruments, are one or two orders of magnitude greater than those reported from ambient measurements.^28,62^ This is mostly likely due to differences in the proximity to the fire and plume dilution processes.

The average EF_N_ values, shown in Table 4, were in agreement with the singular laboratory study that has considered peat emission factors,^37^ and the limited smouldering literature values associated with other types of biomass burning.^60,63,98,99^ To date, no field studies have quantified EF_N_ for smouldering peat fires. The EF_N_ values will be dependent on the peat type, the ignition protocol, the burn temperature and efficiency, sampling dilution, and particle losses within the sampling system but are expected to be a conservative estimate as our measurements do not account for any atmospheric ageing and secondary organic aerosol nucleation processes.^37^

### 3.3 Particle carbon content

The high contribution of OC to total carbon (TC) (96 ± 3%) and the OC : EC ratio (range: 181 to 212) are typical for smouldering or low-temperature combustion fires of various biomasses,^24,73,90,100^ and are strongly indicative of pyrolysis processes. Conversely, for flaming or mixed phase burns the OC : EC ratio decreases with the increase in EC emissions.^100^ The variation in peat type, combustion dynamics, and sampling location, as well as thermal optical method protocols consequently leads to the broad range of OC/EC ratios within the literature, as shown in Table S-6 in the ESI.† Visual inspection of the particle samples on the filters further supports the high OC content: the filters were coated with bright yellow somewhat-oily particles as shown in Fig. S-2 in the ESI.† Additionally, the percentage of OC as a function of the total particle mass (76%) was consistent with values stated in laboratory and field studies as shown in Table S-6 in the ESI.†

### 3.4 Particle metal content

Metals can be displaced during the combustion of peatlands either through vaporisation or emitted as fly ash particles,^101^ however the relatively low proportion of the total particle mass that was attributed to metals (3.1 ± 0.5%), compared to other quantified species such as OC and the ash remaining in the residue, indicated minimal transfer of metals to the particle phase.

Al, Fe, K, and Mg were the most abundant trace metals detected in the particle samples. Together the metals accounted for approximately 85% to the total particle-bound metal mass detected. The high proportion of crustal metals was expected due to the high abundances of the metals in peat core samples, and the potential for alkali metals, present as salts, to be released at the low temperatures associated with smouldering combustion.^102,103^ Other trace metals such as Ba, Zn, and Sr were also detected; however, unlike in previous studies,^32,104^ toxic metals, such as Pb, and Ni were not detected in the particles collected during this studies' Irish peat fires. The metal content of the particles was also found to be lower than previous studies of flaming, high temperature, burns.^57,105^

**Fig. 4:**
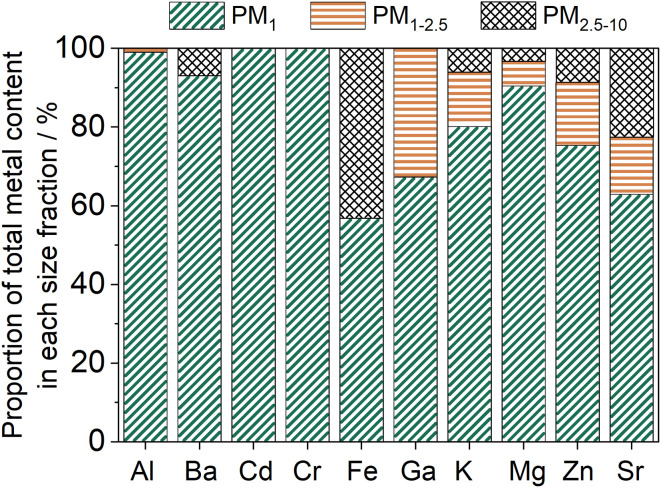
The proportion of individual metals in the three particle samples relative to the total particle-bound mass of each metal.

In this study, approximately 86% of the total quantified metal content resided in the PM_1_ fraction, which aligned with a higher PM_1_ mass in comparison to the other particle size fractions. The contribution of individual metals to the PM_1_, PM_1–2.5_ and PM_2.5–10_ fractions is shown in Fig. 4. Carcinogenic metals, Cr and Cd, were only detected in the one of the PM_1_ samples, whilst Fe was only above the limit of quantification in three of the nine PM_1_ samples. When detected, Fe accounted for around 4% of the PM_1_ total metal mass.

The relative contribution of each metal to the total elemental mass has previously been found to vary depending on geographical location of the peat fire, the temperature of the burn, and number of metals analysed for.^36,105^

**Fig. 5:**
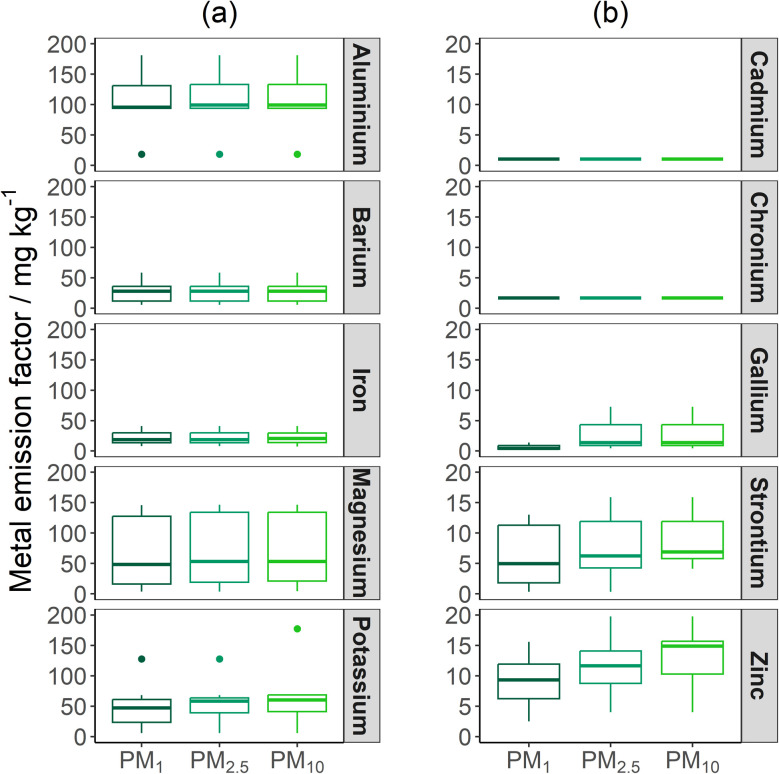
Boxplot of the particle-bound major (a) and minor (b) trace metal EFs for the Irish peat burns. The lower and upper box boundaries represent 25th and 75th percentiles, respectively, whilst the line inside the box represents the median value. Error lines represent the maximum and minimum data, and filled circles represent data that falls outside the extremes of the boxplot (25^th^ and 75^th^ percentile ± 1.5 × interquartile range).

The size-resolved metal EFs for this study are shown in Fig. 5. The particle-bound metal EFs are in agreement with the limited number of studies that have evaluated elemental EFs.^24,36^ Metal EFs, as a function of fuel consumed, are limited in the literature because often CO and CO_2_ emissions are not monitored, or the mass of peat burnt in unknown. To date, metal concentrations and ratios (metal concentration as a function of particle mass) are more frequently reported.^24,29,32,62,92,103,106^ Other biomass burning studies (including forest, savanna, and crop residue fires) have also stated particle-bound metal EFs of similar magnitude to this study.^41,107^

### 3.5 Particle ionic content

Sulfate accounted for 18% of the total ionic mass in the PM_10_ fraction, whilst particle bound ions, NH_4_^+^, K^+^, and Na^+^, constituted around 31% of the total ionic mass for the collected particles in this study, which is in agreement with existing ambient literature.^33,62,92^ Water-soluble Mg and K concentrations were equivalent to less than 80% of the total element concentration detected by ICP-OES.

**Fig. 6:**
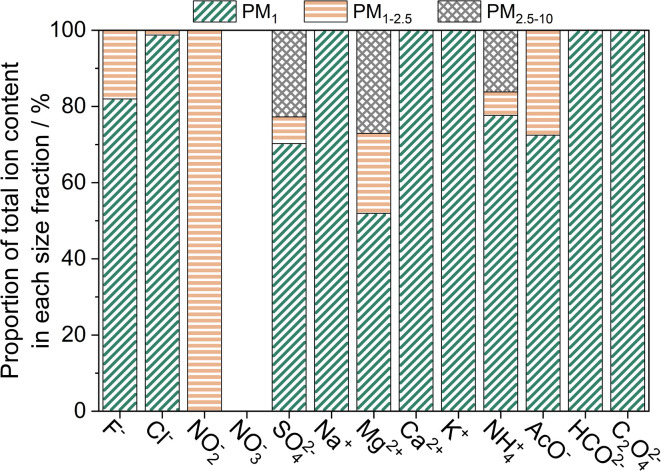
The proportion of individual ions in the three particle samples relative to the total particle-bound mass of each ion.

Size-resolved analysis also identified quantifiable concentrations of water-soluble F^−^, Cl^−^, and NO_2_^−^ ions, as shown in Fig. 6. The detected ions were found to predominantly reside in the particle samples with a *d*_a_ less than 1 μm (89 ± 3% of total ionic concentration). This is in agreement with a previous temperate peat study which identified over half of the ionic species resided in the fine particle fraction.^33^ Ions such as NH_4_^+^, Mg^2+^, SO_4_^2−^ and NO_2_^−^ were also detected in particle samples with a *d*_a_ greater than 1 μm, whilst ionic species, such as Na^+^, K^+^, and Ca^2+^ were only detected in samples with a *d*_a_ less than 1 μm, as shown in Fig. 6.

Low molecular weight acids (acetate, oxolate, and formate) accounted for around 0.6% of the total particle mass detected in the particle samples. Acetate and formate accounted for 13 ± 6% and 14 ± 9% of the total ionic mass, whilst oxolate accounted for less than 10% of the total ionic mass. The higher abundance of monocarboxylic acids than the dicarboxylic acid (oxolate) is indicative of predominantly primary combustion emissions.^62,108,109^ A larger ratio of oxolate to formate/acetate would have indicated greater levels of secondary photochemistry and liquid-phase oxidation.^110^

**Fig. 7:**
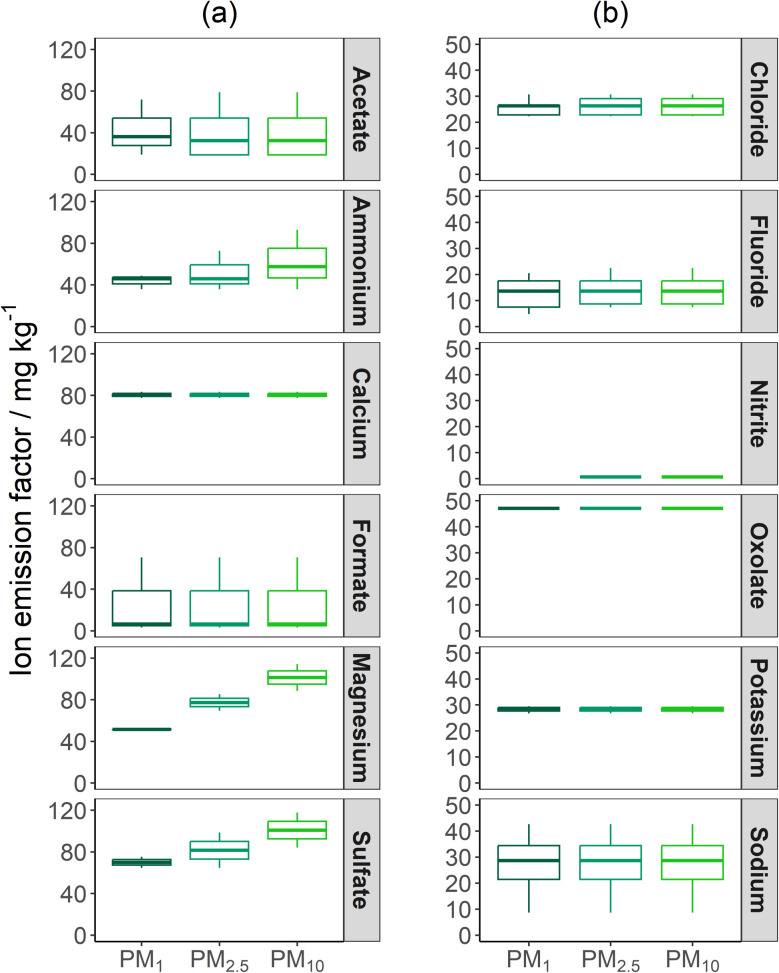
Boxplot of the particle-bound major (a) and minor (b) ion and low molecular weight acid EFs for the Irish peat burns. The lower and upper box boundaries represent 25th and 75th percentiles, respectively, whilst the line inside the box represents the median value. Error lines represent the maximum and minimum data, and filled circles represent data that falls outside the extremes of the boxplot (25^th^ and 75^th^ percentile ± 1.5 × interquartile range).

The size-resolved particle ionic EFs are shown in Fig. 7. For the first time ionic EFs have been reported for Irish peat burns, and only a limited number of temperate peat fire emission studies have determined the particle ionic EFs. The particle-bound EFs, in particular Cl^−^, NH_4_^+^, K^+^, and SO_4_^2−^ EFs, were in agreement with literature values for temperate peat fires.^33^

Other peat fire literature has identified the distribution of ionic species as a function of particle size varies with peat type, geographical location, combustion conditions and aging of plume.^23,33,62^ Literature has identified Na^+^ and Cl^−^ as the most abundant ionic species, whilst the proportions of F^−^, K^+^, SO_4_^2−^, NO_2_^−^, NO_3_^−^, Mg^2+^, and Ca^2+^ ions has shown to vary. Alongside the variations in peat soil and combustion dynamics, the gaseous emissions and gas-to-particle conversion after combustion influence the concentrations of SO_4_^2−^, NH_4_^+^, and other nitrogen-based ions. Therefore, as the particles were collected close to source in this study the concentrations of SO_4_^2−^, NH_4_^+^, and other nitrogen ions are likely to be lower than that measured in the ambient environment.

### 3.6 TEM and Raman analysis

TEM images of the particle samples showed an array of particle sizes; the size distribution of the 60 analysed particles is shown in Fig. S-3 in the ESI.†

**Fig. 8:**
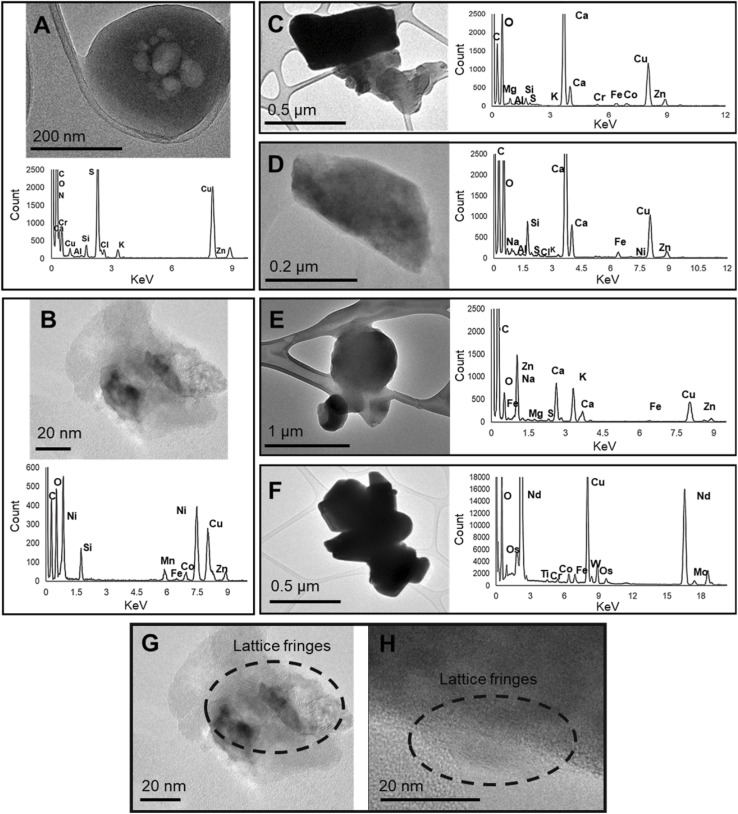
TEM micrographs and EDS spectra for a range of different particles emitted from smouldering peat. The STEM-EDS maps of the particles are presented in Fig. S-4 to S-10 in the ESI.† C and Cu peaks also arise from the carbon film, copper TEM grids. Micrographs and EDS spectrum of: (A) a particle that contains a concentration of S and O, as well as other trace elements (Cr, Ca, Al, Si, Cl and K) and has a porous core; (B) a particle with a crystalline structure (lattice fringes shown in (G)) and consists of a high concentration of C, O and other trace elements (Ni, Si, Mn, Fe, Co, and Zn); (C) particles consisting of Ca, O and other trace elements (Na, Al, Si, S, Cl, K, Fe, Ni and Zn); (D) a particle with lattice fringes (shown in (H)) and consisting C, O and other trace elements (Ca, Fe, K, Mg, Na, S, and Zn); (E) a cluster of particles consisting of C, O, Fe, Zn, Na, Mg, S and Ca; (F) dense irregular particles held in an organic matrix, shown in Fig. S-8 in the ESI,† and consists of O, Os, Ti, Cr, Co, Fe Nd, Mo and W.

**Fig. 9:**
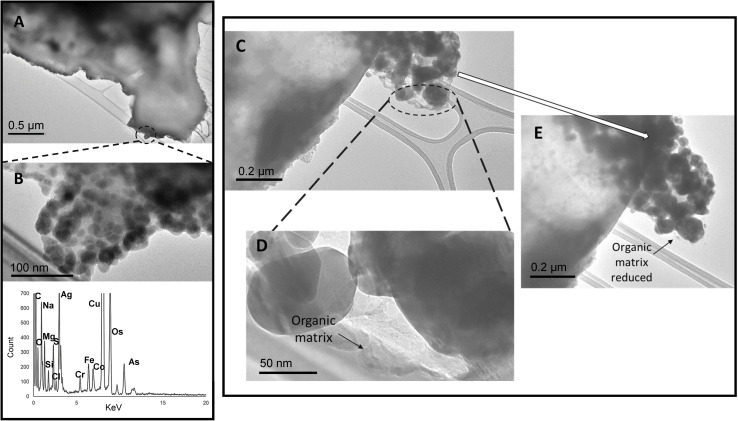
TEM micrographs and EDS spectra of clusters of particles held in an organic matrix. The STEM-EDS maps of the particles are presented in Fig. S-10 in the ESI.† C and Cu peaks also arise are from the carbon film, copper TEM grids. Micrographs and EDS spectrum of: (A) a carbonaceous particles, (B) higher magnification image of the edge of the particle in (A) and corresponding EDS spectrum, (C) another region of the particle shown in (A), which shows a cluster of particles, (D) higher magnification image of the oval region in (C) showing it is made up of an organic matrix and particles, (E) same segment of particle shown in (C) after 5 minutes exposure to electron beam.

The complexity of the particle morphologies and composition are shown in Fig. 8 and 9. EDS maps of the particles in Fig. 8 are shown in Fig. S-4 to S-10 in the ESI.† Many of the particles had irregular and non-spherical morphologies, for example, particles B–F in Fig. 8. Particles A and E in Fig. 8 appeared to spread over the grid, indicating that these particles were hydrated, had a low viscosity, or a low surface tension.^111–113^ Particles A and E are consistent with those collected near to biomass fires (fresh samples), whilst aged-smoke particles (further away from fire) have previously been found to be spherical and have become known as tarballs.^111,113,114^ Tarballs are defined in literature as spherical, amorphous, not aggregated with other particles or have inclusions, stable under the electron beam, and are likely form from gas-phase nucleation or secondary processes within the primary particles as the plume ages.^111,113,114^

The particles, shown in Fig. 8, had a varied composition and were composed of a range of different elements, including Al, Fe, Mn, Ca, Ni, K, Zn, S, Cr, and Nd. The composition of the particles in Fig. 8(B and D) was consistent with previous findings of fly ash or mineral dust particles in biomass burning plumes.^115^ The presence of metal salts in biomass burning particles, such as potassium sulfate, has also been reported.^113^ Organic sulfur is also common in peatland soils,^116^ which can be emitted in the form of sulfur dioxide and undergo heterogenous oxidation with nitrogen oxide on the surface of particles to formulate the sulfur-rich particles in Fig. 8(A).^117,118^

Raman spectra, shown in Fig. S-11 in the ESI,† further confirmed the presence of Fe, through the identification of iron oxides such a goethite, ferrihydrite, and haematite. The transformation of these crystal phases is dependent on temperature and pH, and may be catalysed by cations such as Zn and Ni.

The extraction effectiveness of particle-bound metals for ICP-OES analysis was likely dependent on the form Fe was present in, such as iron-oxides or iron-aluminosilicates.^119,120^ As a result the total elemental content may not have been detected by ICP-OES analysis, because the HNO_3_ extraction protocol would have been unable to extract elements such as Al, K, Ca, Fe from complex silicate structures.^121–123^ Higher extraction of Fe was found in literature when HNO_3_ and hydrofluoric acid were used, indicating Fe was present in the particles as Fe-silicates.^27^

One of the most abundant types of particles on the TEM grids were organic carbon particles with inorganic inclusions (Fig. 9). These particles did not have distinct morphologies and instead clusters of particles appeared to be held in an organic matrix. The composition of the particles was predominantly C and O with minor elements such as Al, Fe, Na, and Co, which may exist in sulfate or chloride salts (see Fig. S-10 in the ESI†). The inorganic inclusions, and the fact that the particle organic matrix was unstable during exposure to the electron beam (Fig. 9) indicates that the particles were not tarballs or black carbon (aggregates of soot particles).

The crystallinity of the carbonaceous particles with a diameter greater than 1 μm can be semi-quantitatively evaluated using Raman spectroscopy. The G band (∼1580 cm^−1^) can be attributed to the stretching vibration mode with E_2g_ symmetry in the aromatic layers of the graphite crystalline, whilst the D_1_ band at 1350 cm^−1^ is only active in the presence of disordered carbon and heteroatoms.^124–126^ In perfect crystalline carbonaceous material only the G band is present in the first order region.^126^ The Raman spectra for the particles are shown in Fig. S-11 and S-12 in the ESI.† Both the D and G bands were present for the particles investigated, indicating the presence of amorphous carbon. This is supported by a broad peak between 1500 and 1600 cm^−1^, as shown in Fig. S-12 in the ESI,† consistent with previous literature.^127^ Comparatively, Fig. S-11 in the ESI† displays particles which have distinct D_1_ and G bands. The D_1_/G intensity ratio, known as the *R*_1_ ratio,^128^ was also calculated for the particles and presented in Table S-7 in the ESI.† The relative intensity of these bands provides a means of assessing the degree of crystallinity; the larger the *R*_1_ ratio, the greater the amount of disordered carbon within the carbonaceous particles, whilst smaller ratio values alongside smaller full width at half maximum (FWMH) values indicate greater levels of graphitisation.^124^ For ∼80% of the particles analysed, the *R*_1_ ratio indicated less graphitised carbon structures and greater levels of disorder.

**Fig. 10:**
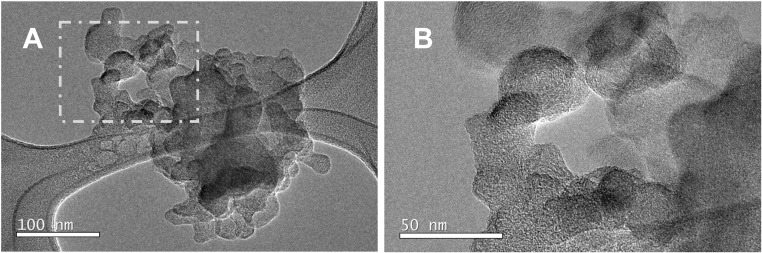
Micrographs of a soot agglomerate identified on the TEM grid: (A) the entire soot agglomerate, and (B) graphitic layers within the soot agglomerate (top left corner of micrograph shown in (A)).

Our TEM analysis found relatively few black carbon particles (*e.g.*Fig. 10), providing further evidence that black carbon does not make up a significant fraction of particles emitted from smouldering peat fires, in agreement with literature.^129^ Flaming fires are known to emit greater levels of black carbon, in the form of soot aggregates.^113,130^

## 4 Implications

This study used the combination of aerodynamic and mobility diameter measurements for particle mass- and number-weighted concentrations. The average PM_2.5_ mass concentration determined was greater than the 24 h mean air quality guideline values (15 μg m^−3^) published by the WHO, whilst the *in situ* measured number-weighted particle concentration was two orders of magnitudes greater than the WHO's stated high 1 hour concentration (20 000 particles per cm^3^). Fine and UFP can penetrate deeper in the respiratory system than coarse particles, and impact the respiratory system, heart rate variability, and cause mortality.^52,131^ For example, literature has identified that the inhalation of UFP emitted from peat fires can decrease cardiac function and lead to a greater risk of a heart attack, whilst coarse particles can increase lung inflammation.^48^ The comparisons of the aerodynamic and mobility diameter measurements further highlighted the importance of understanding the complex morphology, density, chemical composition, and dynamic nature of the particles emitted during wildfire events. OC was found to constitute 76% of the total particle mass. Greater levels of OC and polar organic compounds in biomass burning particles have been correlated with a higher oxidative potential.^132–134^ Additionally, particle-bound carcinogenic PAHs have previously been found to constituted 0.1% of the total PM_2.5_ OC mass.^17^ The particle-bound PAHs in the peat fire emissions from this study will be reported elsewhere. Metals bound to the fine particle fraction can also be transported deep into the respiratory system and have been associated with adverse health effects.^29^ For example, the uptake of redox active Fe in the body has been linked with hypertension, damage of tissues (such as liver, heart and joints) and neurological disorders such as Alzheimer's and Parkinson's disease,^76^ whereas exposure to particle-bound Zn has also been correlated with inflammation, fibrosis, and pulmonary diseases.^135^ Metal analysis by ICP-OES did not detect carcinogenic particle-bound Pb, and Ni, but TEM particle analysis identified metals such as Ni. The uptake of Ni, Cr(vi), and Cd in the respiratory system can lead to adverse health effects such as respiratory disorders, kidney damage, and DNA damage.^136^ However, there are inconsistencies in the literature as to what contribution metals, such as Fe, Ni, Cr, Mn, and Zn make to the oxidative potential of the emitted particles and the health effects associated with the inhalation of the particles.^27,132^ Consideration of dose, site of deposition, and bioavailability of the metals is important for toxicity studies.^29,137^ However, the very high particle load during peatland fires, as well as redox active metals in the fine particle fraction provide a cause for concern for regional air quality and individuals, such as wildfire firefighters, who may be present in the immediate vicinity of the fires.^29^

Wildfire emission inventories and radiative forcing modelling also suffer from a limited number of particle EFs (mass- and number-weighted) and the possible underestimation of sources of brown carbon.^133^ This study provides near-source number- and mass-weighted particle EFs, as well as specific chemical species EFs for smouldering-only peat fires. Consequently, this study builds on the limited number of temperate peat laboratory studies and provides greater understanding of how peat type and combustion dynamics can influence the release of particles, which could be applied to more complex field measurements and inform future atmospheric modelling work.

Furthermore, microscopy analysis found various Fe mineral phases. The partitioning of the mineral phases is important for assessing the light absorbing capacity of the particles and consequently the radiative effect of the particles emitted.^138–140^ For example, the partitioning of goethite and hematite phases may be important for climate models because they are light absorbers in the shortwave spectrum.^138^

## 5 Conclusions

Simultaneous particle size distribution measurements, alongside offline chemical characterisation allowed for the size-resolved characterisation of particles emitted from smouldering peat fires. This included the evaluation of the *in situ* particle mass and number concentration, morphology, and chemical composition (organic carbon, elemental carbon, metals, and ionic species).

Particles with aerodynamic diameter of less than 2.5 μm contributed a significant proportion (94 ± 2%) of the total particle mass. Organic carbon accounted for 76% of the total particle mass, whilst microscopy and elemental carbon analysis provided only limited evidence of black carbon (<0.5% of total particle mass was elemental carbon). This provided further evidence that the optically active absorbing component of elemental carbon, black carbon, is not a major component of particles emitted from smouldering fires. Microscopy analysis identified an array of particle morphologies, including irregular shaped metal particles, spherical particles with sulfate chemistry, and organic matrix particles with internal particle clusters. Carcinogenic metals, such as Cd and Ni, were also present in some of the ultrafine particles. The size-resolved chemical analysis further found the fine particle fraction consisted of redox active metals such as Fe, as well as ionic species such as sulfate. As a result of the high *in situ* fine particle load, alongside 86% of the total metal mass residing in the smaller size fraction (less than 1 μm), exposure to the near source emissions could be a cause for concern for regional air quality and human health.

With the high risk and severity of smouldering peatland fires, especially in a warming climate, this study will inform research into the causal mechanisms that lead to adverse health effects if the particles are inhaled, as well as the particles direct and indirect climate forcing. Further research is required to investigate the variability in the size-resolved particle physicochemical characteristics and toxicity from a variety of smouldering fires as a function of plume age. Additionally, further work is required to characterise mineral phase partitioning, direct radiative effects, and cloud condensation nuclei activity.

## Data availability

Data for this article, including microscopy spectral data files, Raman spectroscopy data files, particle mass, number and feret diameter size distribution data files, and particle-bound metal and ion concentration and emission factor data files are provided as ESI.†

## Author contributions

ALW: conceptualisation, methodology, investigation, writing – original draft, and visualisation. WC: methodology, investigation, and writing – review & editing. YH: methodology, investigation, and writing – review & editing. MC: investigation, writing – review & editing. GR: conceptualisation, methodology, resources, writing – review & editing, supervision, and funding acquisition. AEP: resources, and writing – review & editing. GF: resources, writing – review & editing, and supervision. MEJS: conceptualisation, methodology, resources, writing – review & editing, supervision, and funding acquisition.

## Conflicts of interest

The authors declare that they have no conflict of interest.

## Supplementary Material

EA-005-D4EA00124A-s001

EA-005-D4EA00124A-s002
