# Supplementary material for: Particles emitted from smouldering peat: size-resolved composition and emission factors
Source: Environ Sci Atmos. 2025 Jan 20;5(3):348–66. doi: 10.1039/d4ea00124a (PMC11827554; doi:10.1039/d4ea00124a)
Supplement: EA-005-D4EA00124A-s002 [file EA-005-D4EA00124A-s002.pdf]

Supplementary Information (SI) for Environmental Science: Atmospheres.
This journal is © The Royal Society of Chemistry 2025

SUPPORTING INFORMATION FOR PARTICLES EMITTED FROM SMOULDERING PEAT: SIZE-RESOLVED COMPOSITION AND EMISSION FACTORS

A L. Wilson[1], Wuquan Cui[2], Yuqi Hu[2,3], Marta Chiapasco[4], Guillermo Rein[2], Alexandra E. Porter[4], Geoff Fowler[1], and Marc E.J. Stettler[1]

[1]Department of Civil and Environmental Engineering, Imperial College London, London, United Kingdom

[2] Department of Mechanical Engineering, Imperial College London, London, United Kingdom

[3] Sichuan Fire Research Institution of Ministry of Emergency Management of China, Chengdu, China

[4] Department of Materials, Imperial College London, London, United Kingdom

1.0 Particle measurements

The number of peat burns that were monitored for particle number concentration, as well as the total number of particle collections made across the 12 peat burns, are summarised in Table 1.

Table S-1. A summary of the measurements taken during the Irish peat burns.

Instrument or Sampler	Measurement or collection repeats
Scanning Mobility Particle Sizer (SMPS)	Continuous measurement, 5 burns in total
Electrical Low-Pressure Impactor (ELPI)	Continuous measurement, 7 burns in total
Micro-Orifice Uniform Distribution Impactor (MOUDI)	1 collection per burn (6 burns in total)
Dekati PM_{10} Impactor	13 filter collections over 10 burns<br>(6 filter samples taken forward for IC analysis and 9 for ICP-OES)
Thermophoresis Sampler	1 collection per burn (2 burns in total)
Filter collection using sampling pump	1 collection per burn (2 burns in total)

1.1 Number concentration measurements

The number concentration of particles per stage of the ELPI, *i,* was calculated using Equation 1:

$$C_i = \frac{I}{P \cdot n \cdot e \cdot Q} \quad (1)$$

where C_i is the number concentration of the particles, P is the penetration expressed through the charger, n is the number of charges per particles, e is the charge of an electron, and Q is the flow rate of the ELPI.[1] The particle density was assumed to be 1 g cm^{-3} for the aerosol-charging efficiency calculation because no well-established effective density has been derived for particles emitted from this source.

2.0 Combustion dynamics

The smouldering combustion dynamics for each peat fire were monitored. This included the peat mass lost over the course of the fire, the carbon dioxide and carbon monoxide emissions, and the temperature profiles. An example of one of the peat fires and associated dynamics is shown in Figure S-1.

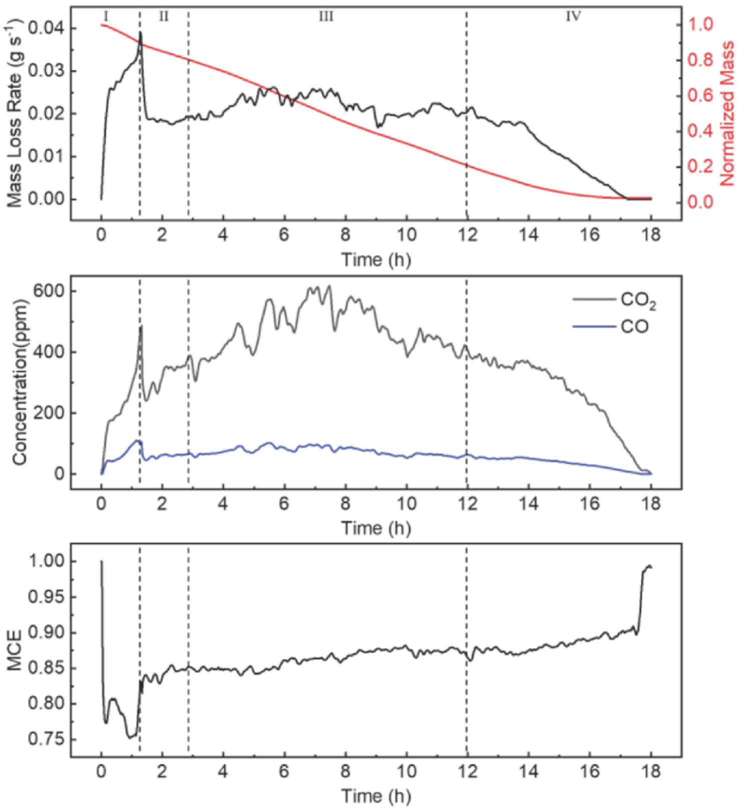

Figure S-1. An example of the combustion dynamic for smouldering peat, including the normalised mass (remaining mass over original mass), mass loss rate, carbon dioxide, carbon monoxide, and the modified combustion efficiency (MCE) profiles. Literature smouldering MCE values are between 0.65 and 0.85. The (I) ignition stage, (II) growth stage, (III) steady stage, and (IV) burn out stage of the laboratory smouldering fires.

3.0 Quantification of error

The uncertainty, standard deviation, and standard error associated with the MOUDI and PM_{10} Impactor filter gravimetric analysis was calculated and is shown in Table S-2 and Table S-3.

Table S-2: The average mass, standard deviation, standard error, and propagated uncertainty for the MOUDI mass measurements

MOUDI cut-off point / µm	Average concentration / µg m^{-3}	Uncertainty / µg m^{-3}	Standard Deviation / µg m^{-3}	Standard Error / µg m^{-3}
9.9	177	32.05	261.2	106.6
6.2	201	27.69	226.7	92.6
3.1	603	22.92	159.8	65.2
1.8	1370	22.54	311.2	127.0
1	917	25.88	240.7	98.2
0.55	1057	23.70	583.9	238.4
0.32	681	16.76	314.6	128.4
0.18	408	19.86	183.1	74.8
0.1	363	27.31	243.4	99.4
0.055	252	32.07	168.1	68.6
0.032	146	35.30	231.8	94.6
0.018	214	76.35	292.5	119.4
0.01	155	25.19	247.0	100.8

Table S-3: The average mass, standard deviation, standard error, and propagated uncertainty for the PM_{10} Impactor mass measurements.

Dekati Impactor cut-off point / µm	Average concentration / µg m^{-3}	Uncertainty / µg m^{-3}	Standard Deviation / µg m^{-3}	Standard Error / µg m^{-3}
10	417	12	485	135
2.5	2184	37	1083	300
1	4120	65	2043	567

4.0 Inductively Coupled Plasma–Optical Emission Spectroscopy and Ion Chromatography data

The limit of detection (LOD) and quantification (LOQ) were determined for the analysis of elements and ions by Ion Chromatography (IC) and Inductively Coupled Plasma – Optical Emission Spectroscopy (ICP-OES), respectively and are displayed in Table S-4 and Table S-5.

Table S-4. An example of the LOD and LOQ of each element analysed for by ICP-OES.

Element	LOD / mg L^{-1}	LOQ / mg L^{-1}
Al	0.0061	0.0203
Bi	0.0240	0.0109
B	0.0008	0.0027
Ba	0.0182	0.0607
Cd	0.0086	0.0287
Co	0.0000	0.0100
Cr	0.0260	0.0867
Cu	0.0185	0.0617
Fe	0.0020	0.0067
Mg	0.0074	0.0247
In	0.0330	0.1100
Ga	0.0220	0.0490
Mn	0.0406	0.1353
Ni	0.0080	0.0267
K	0.0047	0.0157
Sr	0.0001	0.0020
Pb	0.0252	0.0840
Zn	0.0067	0.0223

Table S-5. An example of the LOD and LOQ of each ion analysed for by IC.

Element	LOD / mg L^{-1}	LOQ / mg L^{-1}
K^+	0.002	0.500
Ca^{2+}	0.050	0.100
Mg^{2+}	0.010	0.500
Na^+	0.010	0.200
F^-	0.020	0.020
Cl^-	0.003	0.003
SO_4^{2+}	0.015	0.750
NO_3^-	0.010	0.100
NH_4^+	0.020	0.400
NO_2^-	0.100	0.500
Mg^{2+}	0.010	0.500
Ca^{2+}	0.050	0.100
Li^+	0.003	0.060
PO_4^{3-}	0.150	0.750

5.0 Particle carbon composition

Samples collected on quartz filters were analysed for total, organic, and elemental carbon content. The results are shown in Table S-6 for this study and compared to other literature values.

*Table S-6: Comparison of OC and EC results for bulk particle samples (n=2) collected during the smouldering peat fires with previously reported OC/EC values. *Literature that considers temperate peat emissions.*

Particle size	Study type	Reference	OC / g kg^{-1}	OC / % of PM total mass	EC / % of PM total mass	OC:EC
Total PM	Laboratory	*This study*	11 - 41	74.2 - 77.7	0.37 - 0.41	181.4 - 212.8
$PM_{2.5}$	Laboratory	*Black et al.* (ref [2])*	4.3 - 6.3	73 - 89	-	87 - 115
$PM_{2.5}$	Field	*See et al.* (ref [3])	-	21.4 – 46.9	15 - 19.4	2.42
$PM_{2.5}$	Field	*Jayarathne et al.* (ref [4])	12.4 ± 5.4	72 ± 11	1.1	67 ± 26
$PM_{2.5}$	Laboratory	*Iinuma et al.* (ref [5])*	12.8	-	-	13
$PM_{2.5}$	Laboratory	*Lestari et al.* (ref [6])	3.77 ± 0.82	70	1.9	28-75
$PM_{2.5}$	Field	*Fujii et al.* (ref [7])	-	71.0 ± 5.1	2.1 ± 0.5	36.4
$PM_{2.5}$	Laboratory	*Christian et al.* (ref [8])	6.02	-	-	150
Total PM	Laboratory	*Bhattarai et al.* (ref [9])	6.52 - 9.62	-	-	-

Particles emitted from the Irish peat smouldering fires were collected on various filters throughout the steady stage of the fire, examples of the filters post-collection are displayed in Figure S-2.

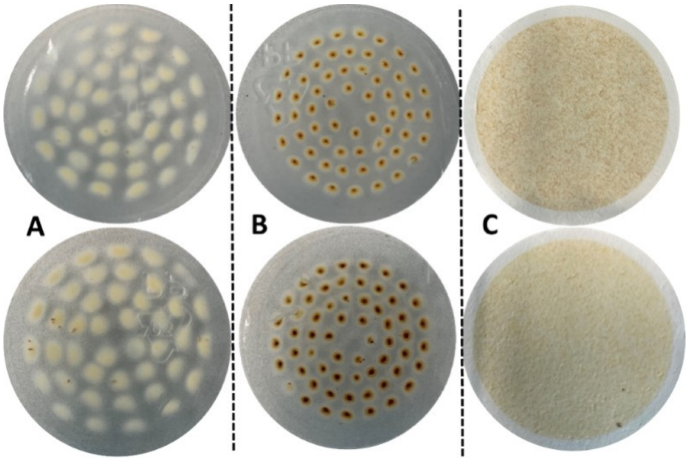

Figure S-2: Example of the particle samples on polycarbonate and Emfab filters collected using the PM_{10} Impactor. Two repeat collections of (A) particles with diameters of 10-2.5 μm, with diameters of 1-2.5 μm, and (C) with diameters less than 1 μm.

6.0 TEM particle size distribution

The size distribution of 60 particles analysed by TEM is shown in Figure S-3.

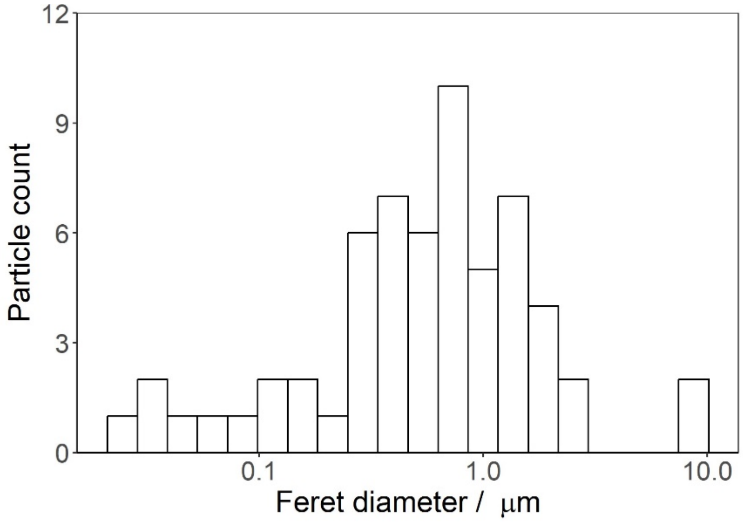

Figure S-3. The size distribution for 60 particles analysed by TEM.

6.0 TEM elemental maps

The Energy Dispersive Spectroscopy (EDS) maps for the particles in Figures 8 and 9, are shown in the Figures below (Figure S-4 to Figure S-10).

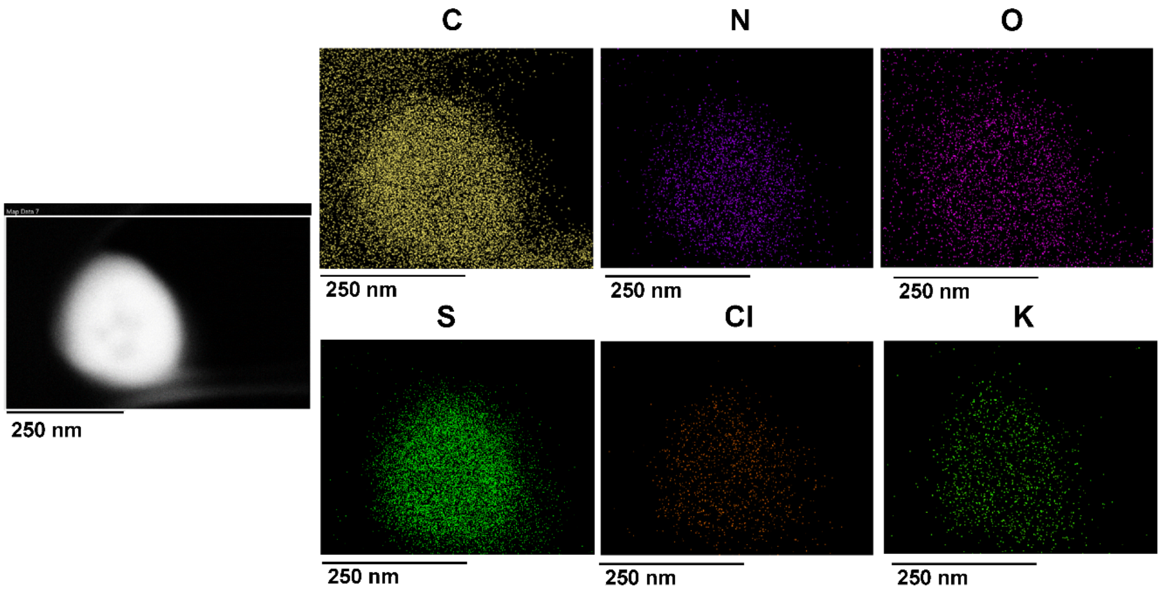

Figure S-4. STEM-EDS maps for particle A in Figure 8. S and O were present in the spherical particle indicating the particle is likely to contain sulphate with other salt grains such as potassium chloride.

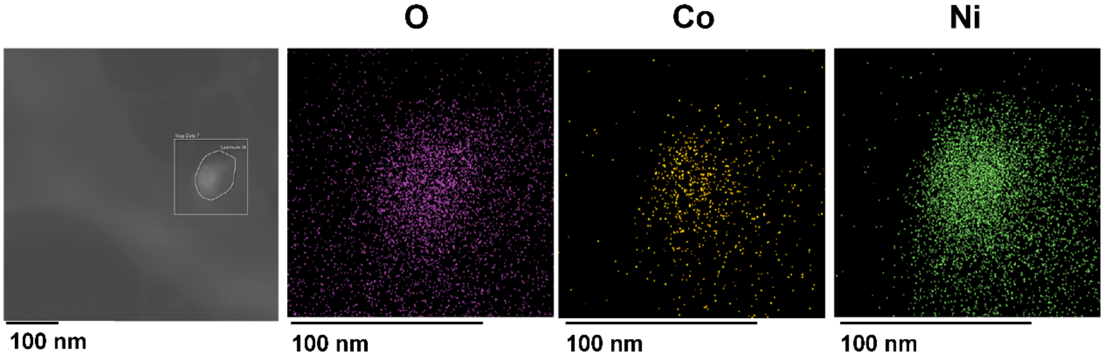

Figure S-5. STEM-EDS for particle B in Figure 8. The resolution of the EDS maps was limited due to the size of the particle, but Ni and O were identified for the particle using the EDS maps.

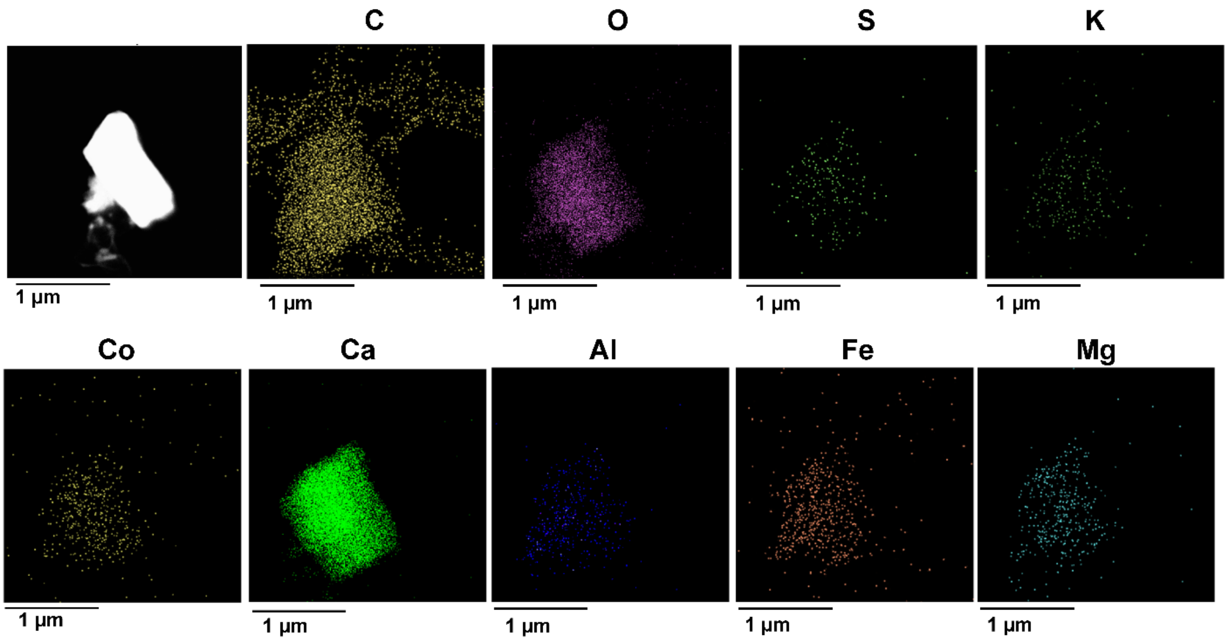

Figure S-6. STEM-EDS for the particles identified in Figure 8 (C). The dense particle has a high weight percentage of Ca and O, whilst the second particle appears to be organic in nature with elemental grains.

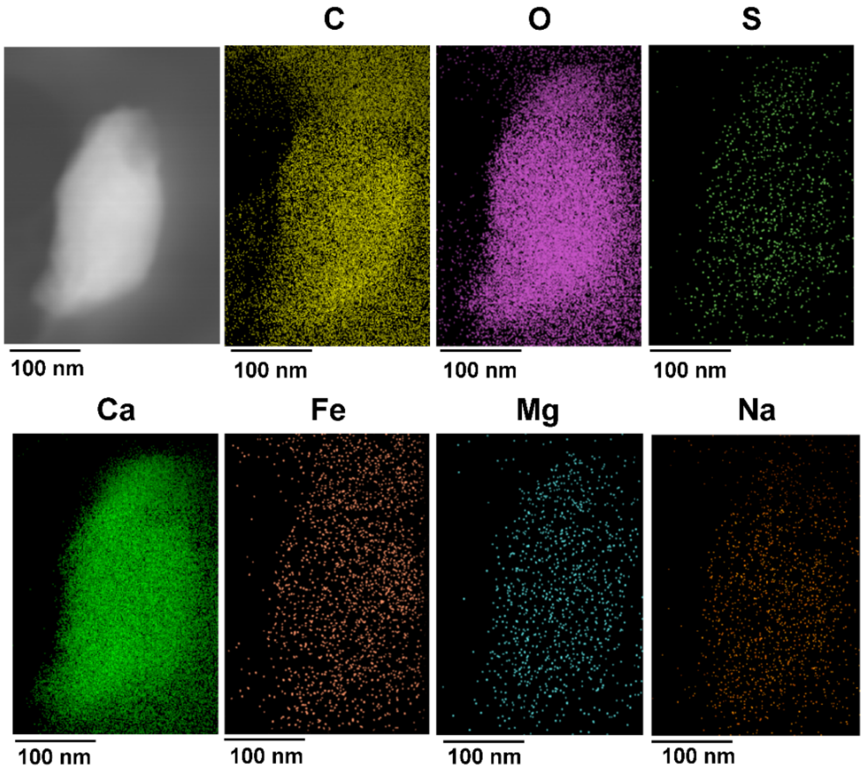

Figure S-7. STEM-EDS maps for particle D in Figure 8. From the EDS maps, the particle likely contains metal oxides including Fe, Mg, and Ca oxides.

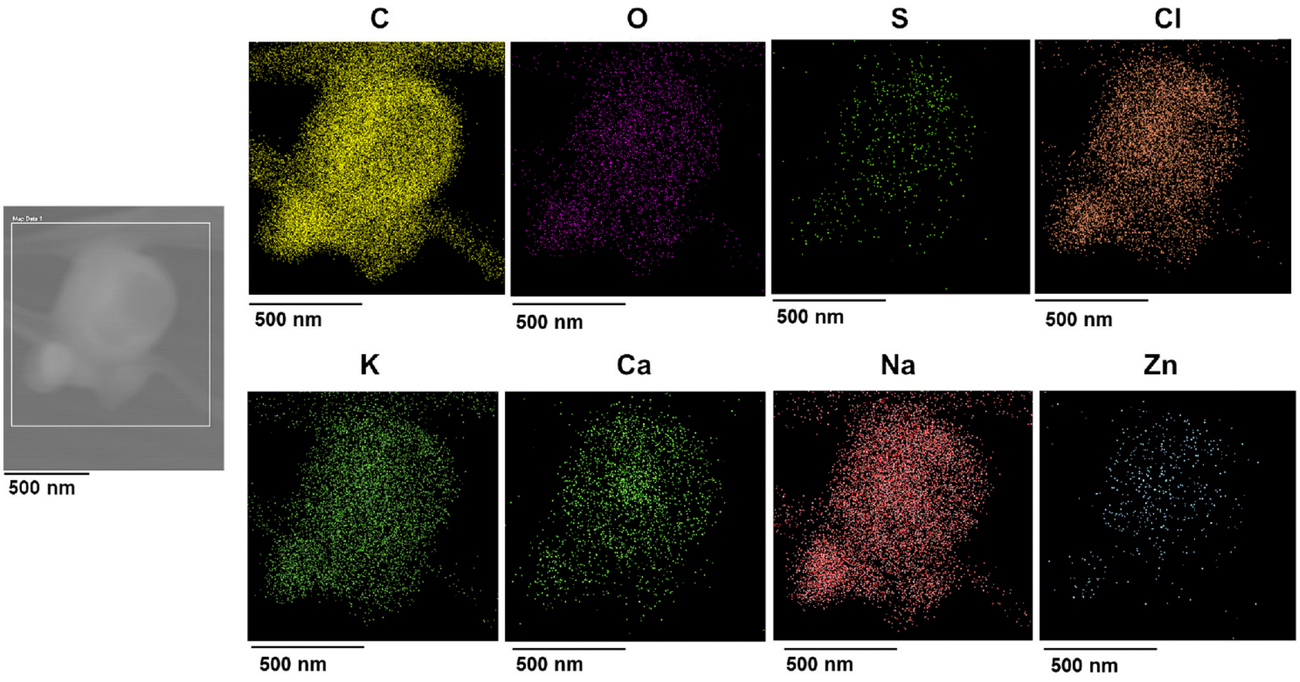

Figure S-8. STEM-EDS maps for the particles identified in Figure 8 (E). The maps indicate the particles are carbonaceous in nature, with the inclusion of metal salts and elements such as sulfur.

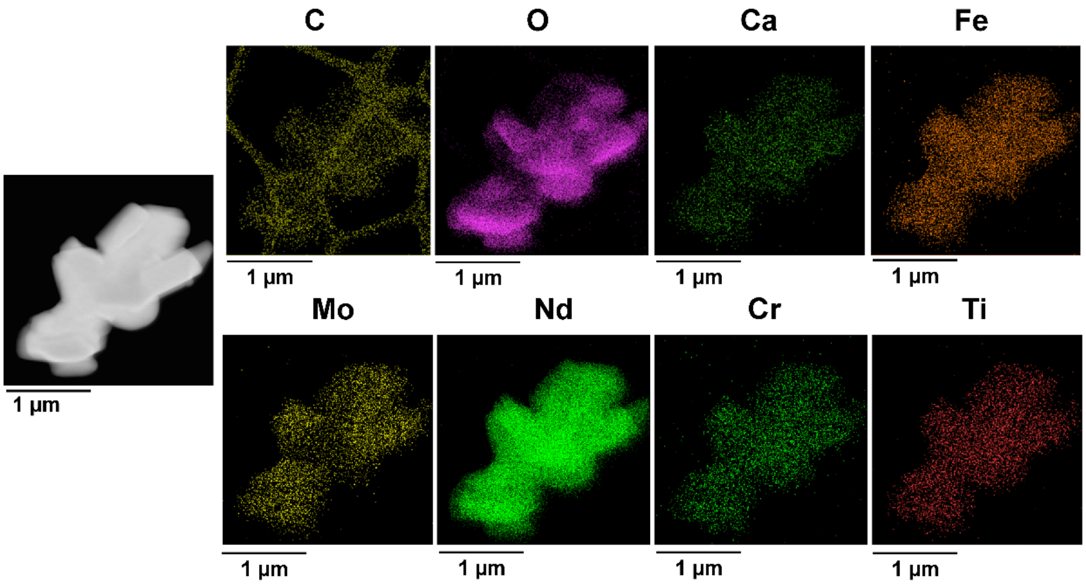

Figure S-9. STEM-EDS maps for the particles identified in Figure 8 (F). The particles appear to be held in an organic matrix and also include metals such as Fe and Nd.

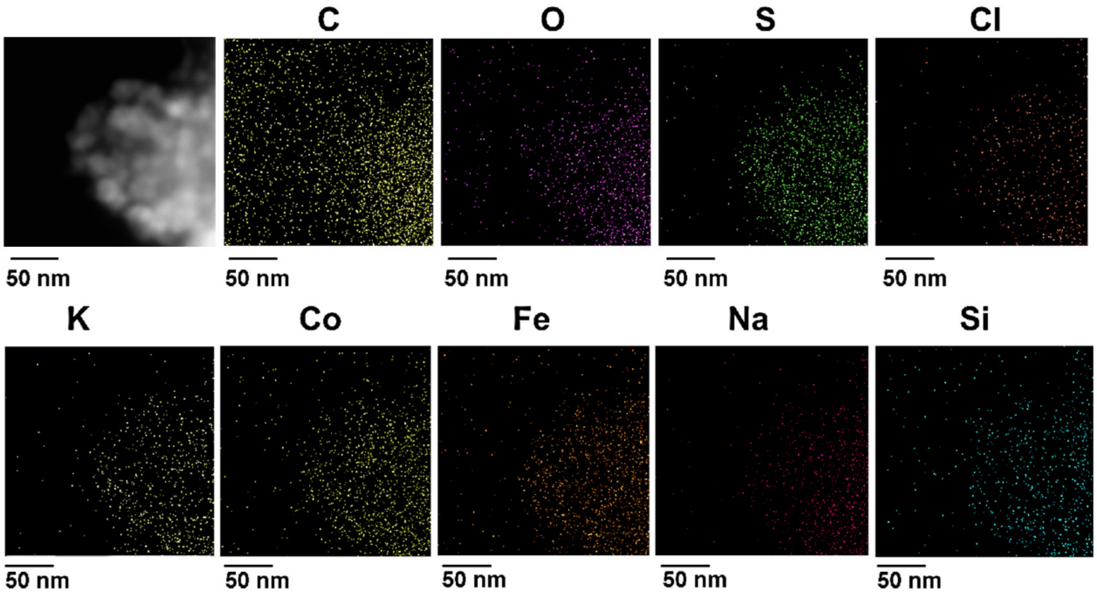

Figure S-10. STEM-EDS maps of the region of the carbonaceous particle displayed in Figure 9. EDS maps indicate the particle's composition is made up of both organic (C and O) and inorganic (K, Cl, Co, Fe, Na, and Si) elements.

7.0 Raman spectra

Particles were analysed by Raman Spectroscopy to investigate the particle phases. Figure S-11 displays the three Fe iron phases that were identified in a particle, whilst Figures S-12 and S-13 show the Raman spectra for the carbon phases of the particles. Table S-7 displays the D_1/G intensity ratio, which can be used to assessing the degree of crystallinity.

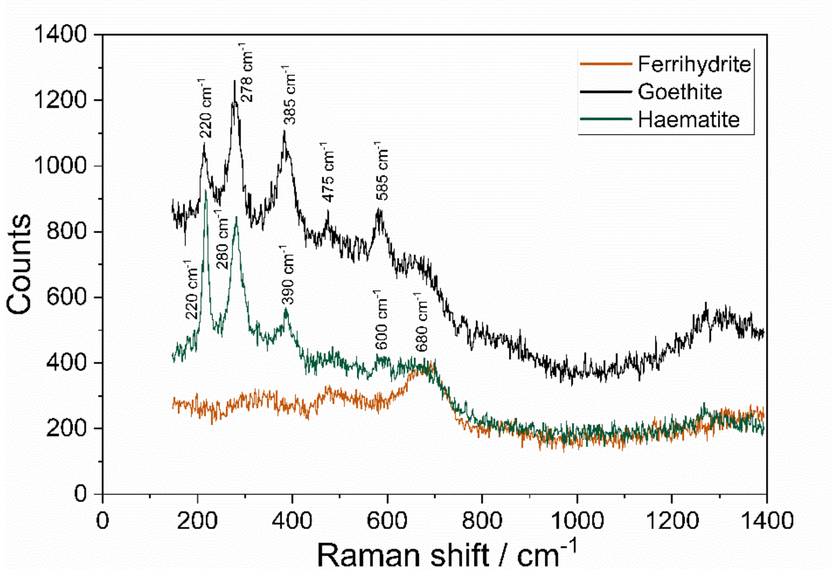

Figure S-11. Raman Spectra taken from three particles emitted during Irish (H) smouldering fires. The peaks are consistent with the Ferrihydrite, Goethite, and Haematite phases.[10]

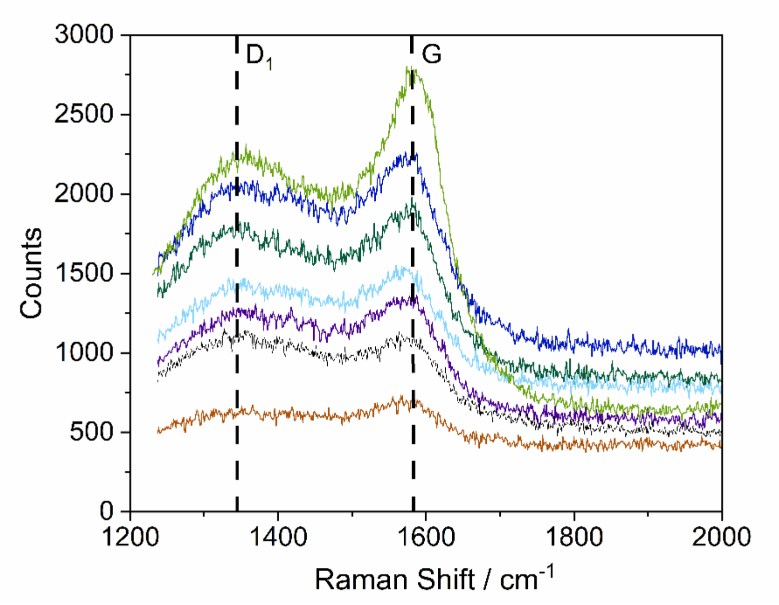

Figure S-12. Raman spectra taken for particles emitted during Irish (H) smouldering fires. The D_1, (defect) band and the G (graphite) band are shown in the figure and can be used as semi-quantitative indicators for the crystallinity and degree of functionalisation of the carbon material within the particles.[11]

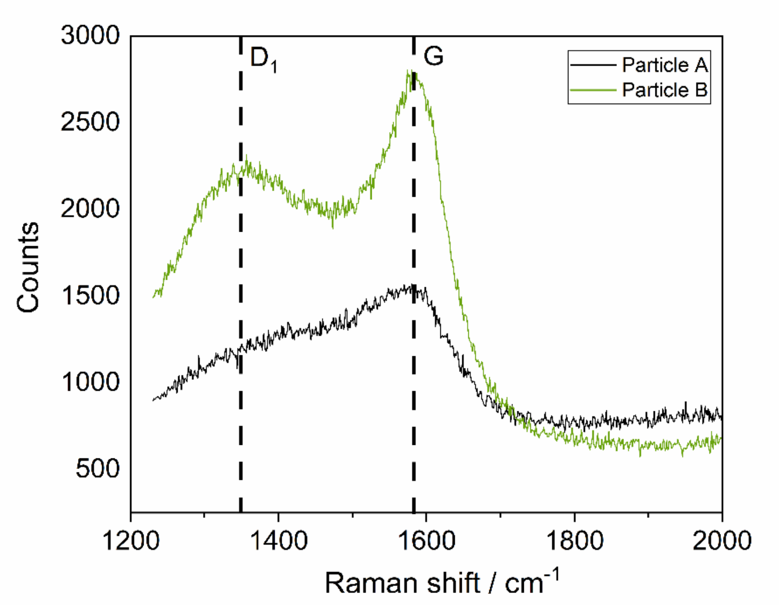

Figure S-13. Example Raman spectra of particles with carbon D_1 and G bands present (A) and amorphous carbon whereby the D and G bands are convoluted (B).

Table S-7. The calculated R1 values for the particles collected during the smouldering Irish (H) fires and analysed by Raman Spectroscopy. The lacey carbon background had a D1/G ratio of 0.87.

Particle Number	D_1/G ratio
1	0.75
2	0.78
3	0.75
4	1.31
5	1.31
6	1.18
7	1.00
8	1.08
9	1.03
10	1.22
11	1.06
12	1.13
13	1.19
14	0.99
15	1.02
16	1.13
17	1.03
18	1.09

References

1 I. Iavicoli, V. Leso, L. Fontana, D. Cottica and A. Bergamaschi, Characterization of Inhalable, Thoracic and Respirable Fractions and Ultrafine Particle Exposure During Grinding, Brazing and Welding Activities in a Mechanical Engineering Factory, *J Occup Environ Med*, 2013, **55**, 430–445.

2 R. R. Black, J. Aurell, A. Holder, I. J. George, B. K. Gullett, M. D. Hays, C. D. Geron and D. Tabor, Characterization of gas and particle emissions from laboratory burns of peat, *Atmos Environ*, 2016, **132**, 49–57.

3 S. W. See, R. Balasubramanian, E. Rianawati, S. Karthikeyan and D. G. Streets, Characterization and Source Apportionment of Particulate Matter < 2.5 μm in Sumatra, Indonesia, during a Recent Peat Fire Episode, *Environmental Science Technology*, 2007, **41**, 3488–3494.

4 T. Jayarathne, C. E. Stockwell, A. Gilbert, K. Daugherty, M. Cochrane, K. Ryan, E. Putra, B. Saharjo, A. Nurhayati, I. Albar, R. Yokelson and E. A. Stone, Chemical characterisation of fine particulate matter emitted by peat fires in Central Kalimantan, Indonesia, during the 2015 El Nino, *Atmos. Chem. Phys*, 2018, **18**, 2585–2600.

5 Y. Iinuma, E. Brüggemann, T. Gnauk, K. Müller, M. O. Andreae, G. Helas, R. Parmar and H. Herrmann, Source characterization of biomass burning particles: The combustion of selected European conifers, African hardwood, savanna grass, and German and Indonesian peat, *J Geophys Res*, 2007, **112**, 1–26.

6 P. Lestari, F. Muthmainnah and D. A. Permadi, Characterization of carbonaceous compounds emitted from Indonesian surface and sub surface peat burning, *Atmos Pollut Res*, 2020, **11**, 1465–1472.

7 Y. Fujii, W. Iriana, M. Oda, A. Puriwigati, S. Tohno, P. Lestari, A. Mizohata and H. S. Huboyo, Characteristics of carbonaceous aerosols emitted from peatland fire in Riau, Sumatra, Indonesia, *Atmos Environ*, 2014, **87**, 164–169.

8 T. J. Christian, B. Kleiss, R. J. Yokelson, R. Holzinger, P. J. Crutzen, W. M. Hao, B. H. Saharjo and D. E. Ward, Comprehensive laboratory measurements of biomass-burning emissions: 1. Emissions from Indonesian, African, and other fuels, *Journal of Geophysical Research D: Atmospheres*, 2003, **108**, 1–13.

9 C. Bhattarai, V. Samburova, D. Sengupta, M. Iaukea-Lum, A. C. Watts, H. Moosmüller and A. Y. Khlystov, Physical and chemical characterization of aerosol in fresh and aged emissions from open combustion of biomass fuels, *Aerosol Science and Technology*, 2018, **52**, 1266–1282.

10 M. Hanesch, Raman spectroscopy of iron oxides and (oxy)hydroxides at low laser power and possible applications in environmental magnetic studies, *Geophys J Int*, 2009, **177**, 941–948.

11 H. S. Leese, L. Govada, E. Saridakis, S. Khurshid, R. Menzel, T. Morishita, A. J. Clancy, E. R. White, N. E. Chayen and M. S. P. Shaffer, Reductively PEGylated carbon nanomaterials and their use to nucleate 3D protein crystals: a comparison of dimensionality †, *Chem Sci*, 2016, **7**, 2916–2923.
